# Additive-free and brine-discharge-free solar-thermal desalination with simultaneous complete mineral mining from ocean water

**DOI:** 10.1038/s41377-026-02315-4

**Published:** 2026-05-27

**Authors:** Luheng Tang, Subhash C. Singh, Ran Wei, Tianshu Xu, Chunlei Guo

**Affiliations:** The Institute of Optics, University of Rochester, Rochester, NY USA

**Keywords:** Solar energy and photovoltaic technology, Laser material processing, Ultrafast lasers, Nanoparticles

## Abstract

Solar-thermal interfacial desalination is a sustainable solution to meet the ever-increasing global freshwater demand. However, when treating actual ocean water, salt accumulation on the evaporator surfaces and brine discharge are major issues limiting the performance and posing environmental concerns. By utilizing a femtosecond laser surface processing technique, we create a multi-functional superwicking black metal (SWBM) panel that can pull a thin water film uphill across its surface, absorb nearly all solar radiation, and most importantly, automatically move the crystalized salts from the active regions to the passive regions for self-cleaning and salt collection. This SWBM serves as an energy-efficient, self-maintained, and additive-free and brine-discharge-free solar-thermal interfacial crystallizer (ABF-STIC) that simultaneously produces fresh water and harnesses nearly all salts directly from ocean water. This self-cleaning effect is attributed to the coffee ring effect and salt creeping, which can be enhanced by deeper and wider grooves, enabling self-cleaning even when treating real ocean water. Our ABF-STIC tracks the sun and operates continuously for a week to purify actual ocean water, achieving an average evaporation rate of 1.76±0.04 kg m^−2^ h^−1^ and salt harvesting rate of 61.74 ± 2.46 g m^−2^ h^−1^ under one sun, corresponding to ~ 74% solar to vapor conversion efficiency and nearly 100% salt extraction.

## Introduction

Freshwater supply is essential not only for life on the planet but also for industrial growth, economic development, and agricultural practices. The continuously growing freshwater demand and depleting natural water supply create a severe water shortage affecting two-thirds of the global population. The escalating global water crisis has created an urgent need to look to the oceans as a source of freshwater. However, current industrial ocean water desalination processes are energy-intensive, have a low water recovery ratio, and generate a high quantity of harmful brine, a mixture of concentrated salt and chemicals. For example, the widely used reverse osmosis (RO) and multi-flash desalination systems have recovery ratios of 0.42 and 0.22, respectively, and produce 0.6 to 6.7 kg of CO_2_ when desalinating one-cubic meter of ocean water^1,2^. Due to the low recovery ratios, the existing desalination systems discharge 58–78% of the inlet water as waste brines, which are either directly released to nearby water sources such as lakes, rivers, ocean shores, or injected underground or spread on land. These practices will negatively impact aquatic lives, land vegetation, and underground water. An ideal practice is to achieve zero brine discharge or commonly termed as zero liquid discharge (ZLD) that would eliminate liquid waste and generate solid salts instead as a byproduct^3–6^.

On the other hand, ocean water contains large quantities, hundreds of times more than on land, of valuable minerals and many of them are scarce and highly valuable. As land-based mining is facing challenges due to the depletion of quality ores, high energy and water consumption, and environmental concerns, mining valuable elements, such as lithium and uranium, directly from ocean water would be highly desirable if it could be integrated in a desalination system^7–13^. Harnessing the valuable minerals from ocean would also reduce the water production cost, as well as minimize environmental concerns associated with the brine discharge. In traditional ZLD systems, ocean water or concentrated brine is spread into a series of evaporation/crystallization ponds to naturally evaporate the water to reach saturation concentration of different salts for crystallization^14,15^. However, this approach is slow, requires large areas of land, and threatens to contaminate underground water from mineral leakage. Therefore, it is highly desirable, both environmentally and economically, to develop a renewable energy powered ZLD desalination system that could rapidly and simultaneously generate freshwater and harness valuable sea salt directly from the ocean water feed.

**Fig. 1:**
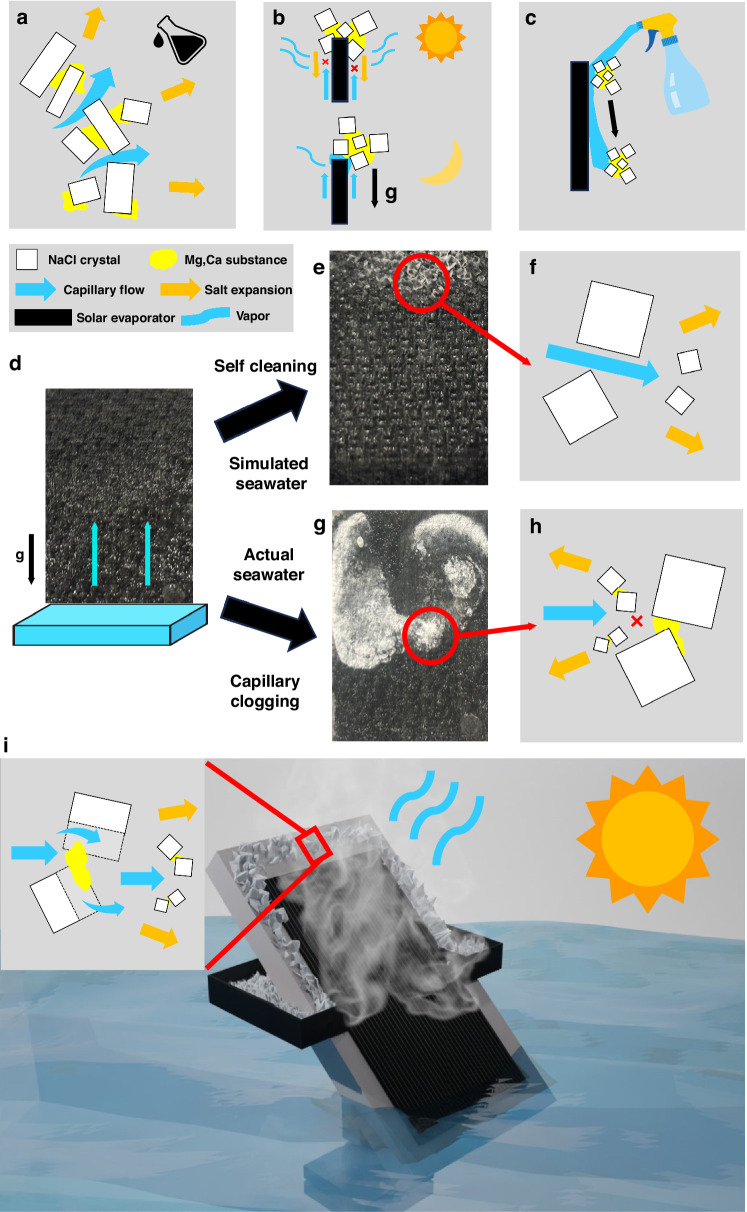
Real ocean water solar-thermal interfacial crystallizer based on super-wicking black metal surfaces. Simulated ocean water only contains NaCl crystals that have many open gaps inside when crystallized, but real ocean water also contains non-porous Mg, Ca substances, which will fill the open pores among NaCl crystals and block the capillary water transport. **a** Schematics showing chemical additives changing the crystal structures and open some pores. **b** Schematics showing nighttime salt dissolution and falling. **c** Schematics showing water spray for cleaning. **d** Commonly-used conventional wicking material. **e** Edgewise crystallization when desalinating simulated ocean water. **f** Schematics showing capillary flow can easily pass through open NaCl crystals for self-cleaning. **g** Capillary clogging when desalinating real ocean water. **h** Schematic diagram showing capillary clogging due to insufficient dissolution due to weak capillary flow, **i** Real ocean water interfacial solar-thermal crystallizer. Inset; a zoomed-in view showing strong capillary flow dissolve salt crystals enabling surface self-cleaning

Recently, solar-thermal interfacial evaporation is shown to be an efficient, multiple times faster than natural processes, and environmentally friendly solution to the global water crisis. Compared to traditional RO-based desalination, solar-thermal interfacial desalination is energy-efficient, grid-free, and sustainable. The interfacial evaporation process requires a combination of materials that can absorb sunlight and wick water. For sunlight absorption, plasmonic nanoparticles^16–18^, carbon-based materials^19–21^, hydrogels^22–24^, and semiconductors^25,26^ were developed. For wicking, a range of water transport networks were developed, including an array of directional micropores^17,18,27^, random porous structures^16,20,22,23,28^, and two-dimensional channels^29^. However, most of the existing interfacial evaporators rely on closed and porous water transport networks, which are inherently prone to clogging^17–19,22–24,29–32^. This issue becomes particularly acute in ocean water desalination, as the active surface area of the existing evaporators will become quickly clogged by crystallized salt, resulting in a decreased performance and eventually cease operation (Supplementary Note S1 for detail of comparison test). Although progresses have been made to crystalize salt on the edge of evaporators, achieving the so-called edge-wise crystallization^13,27,33–37^, actual ocean water has rarely been used due to insufficient dissolving power of capillary flow at salt boundary for existing systems. Therefore, the discussion of the clogging issue was largely avoided in the past by using simulated ocean water, pretreating ocean water by adding chemical additives to change the salt crystal geometries (Fig. 1a), relying on nighttime salt dissolving and falling (Fig. 1b), or mechanical removal^13,27,33–37^. A recent attempt has been made using open capillaries for water transport^38^. However, the evaporator was designed for regular water evaporation rather than salt rejection, making the system still experience extensive salt clogging and require water spraying before reuse (Fig. 1c and Supplementary Note S1). Moreover, this flushing process dissolves the crystallized salt back to the water source, leading to brine discharge. Therefore, none of the previous approaches can resolve the fundamental issues of salt clogging and usually require water pretreatment or leaving salt accumulation on the active area for a period of time before removal, thus reducing the effective operation time.

To appreciate the challenge of desalinating real ocean water, we conduct a comparative evaporation study by using simulated ocean water vs. real ocean water under 1-sun irradiation. The evaporator here is made of commonly-used conventional wicking material by using hydrophilic porous fibers coated with black paint (Fig. 1d). When using simulated ocean water, salt crystallizes on the edge of the wicking material, which shows similar performance to most previous studies (Fig. 1e). This is because NaCl crystallizes in a cubic structure, which leaves interconnected open pores between the crystals. These pores allow capillary flow to pass through the crystal matrix and enable outward crystallization (Fig. 1f). Therefore, the evaporator remains self-cleaning and exhibits stable evaporation. However, the same wicking fibers fail to move salt to the edge when using real ocean water **(**Fig. 1g**)**. We see a clear salt crust formed over the evaporator surface; this crust formation is due to the presence of less soluble minerals in real ocean water, particularly MgSO_4_ and CaCO_3_, which form non-porous, hard and crusty substance between NaCl crystals (Fig. 1h)^34^. Capillary flow with weak dissolving power will be obstructed by salt crystals, leading to backward crystallization and ultimately resulting in clogging. Since ocean water is the primary water source for solar thermal desalination in real-world applications^39^, successful demonstration of real ocean water desalination with stable performance has a huge practical significance.

Here, we demonstrate an energy efficient, solar trackable, self-maintained, and additive-free and brine-discharge-free nanostructure-based direct interfacial solar-thermal crystallizer (ABF-STIC) that simultaneously produces fresh water and harnesses a range of valuable salts from real ocean water (Fig. 1i). These unique features are made possible by utilizing a multi-functional nanostructured superwicking black metal (SWBM) panel that can pull a thin water film uphill across its surface, absorb nearly all solar radiation, and push the crystalized salts from the active regions to the passive regions for self-cleaning and salt collection. In this study, we show that with a proper optimization of the surface structures, the evaporator surface will enable continuous outward salt expansion, thereby enabling self-cleaning when treating real ocean water (Inset of Fig. 1i). The ABF-STIC can also track the sun to maximize solar flux and desalination performance. Our ABF-STIC is used to purify actual ocean water from Pacific, Atlantic, and Indian oceans and operates continuously over weeks without maintenance, achieving an average evaporation rate of 1.76 ± 0.04 kg m^−2^ h^−1^ and salt harvesting rate of 61.74 ± 2.46 g m^−2^ h^−1^ under one sun, corresponding to ~74% evaporation efficiency based on the bulk water vaporization enthalpy and nearly 100% salt extraction. Unlike previous devices, our one-step process desalinates ocean water with zero-liquid-discharge and without any additive, thus realizing a truly sustainable and environmentally-friendly solution for desalination and salt extraction.

## Results

### Superwicking black metal (SWBM) design strategy and material fabrication

For a solar thermal interfacial water evaporation system, the evaporation performance is optimized when all the supplied water gets evaporated, ${\dot{m}}_{{evap}}={\dot{m}}_{{tran}}$, where ${\dot{m}}_{{evap}}$ and ${\dot{m}}_{{tran}}$ are water evaporation and transportation rates, respectively. If ${\dot{m}}_{{tran}}$ is larger than ${\dot{m}}_{{evap}}$, excessive water will remain in the water transport network and lead to thermal loss. On the other hand, if ${\dot{m}}_{{tran}}$ is smaller than ${\dot{m}}_{{evap}}$, dry spots can form on the evaporator. For evaporating distilled water, failure to maintain the water supply and evaporation balance only decrease the evaporation performance. However, when evaporating water with a high solute concentration, such as actual ocean water or brine, ${\dot{m}}_{{tran}}$ needs to be larger than ${\dot{m}}_{{evap}}$; otherwise, the dissolved salt will crystallize on the evaporator surface once the solution reaches above the saturation concentration^38^. Consequently, the crystalized salt will degrade the solar absorption and water transport, increase the evaporation resistance, and reduce the overall performance of the evaporator. We consider the SWBM surface that absorbs light and wicks water as the active region while the surrounding region as the passive region. Salt crystallization on the evaporator surface can be avoided by speeding up the water transport, diluting the active region concentration to below the saturation concentration, and pushing the saturated salt solution towards surrounding passive regions. Therefore, SWBM needs to be carefully designed to balance water transport, optical absorption, and salt accumulation.

**Fig. 2:**
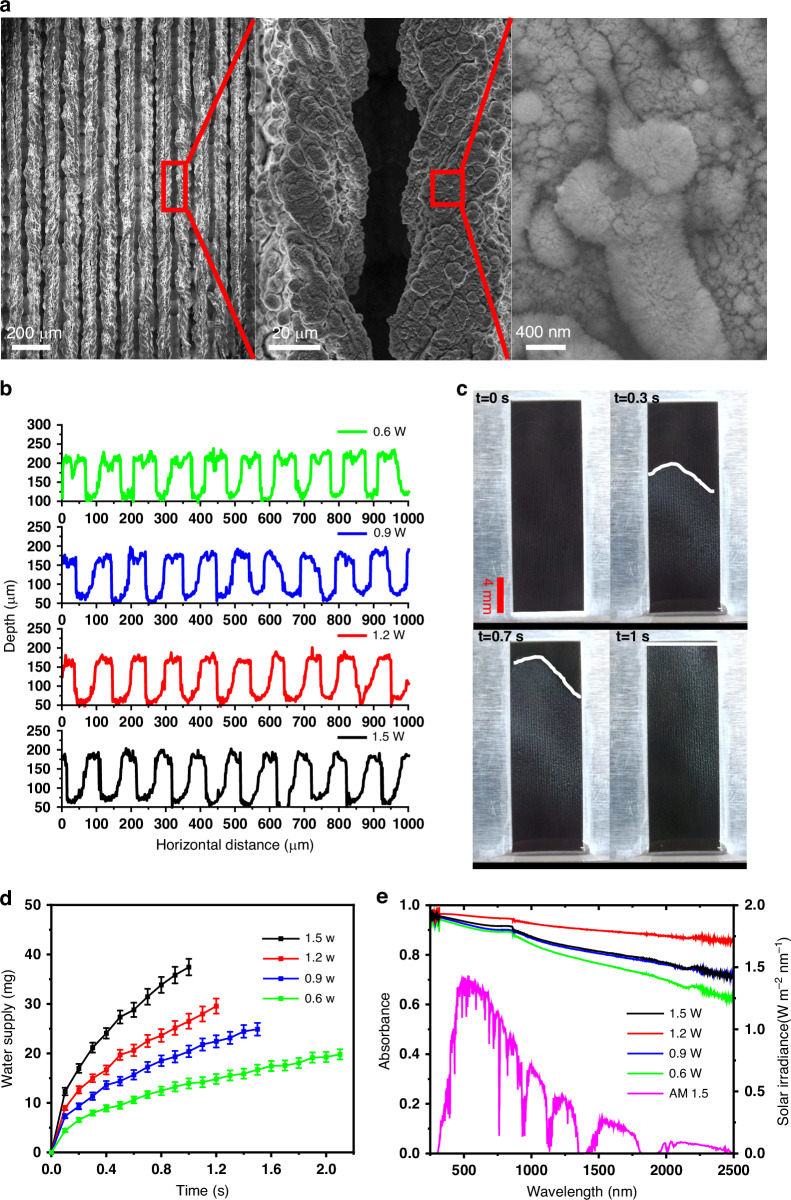
SWBM surface morphology, wettability, and optical absorption measurements. **a** SEM images of the nanostructured SWBM surface under different magnifications (scale bar left to right, 200 μm, 20 μm, and 400 nm). **b** Surface profile of different SWBM samples. **c** Snapshots of water wicking dynamics on the surface of a vertically mounted SWBM sample. White line shows the position of the water wetting front (W_front_). **d** The amount of water wicked onto different SWBM surfaces. Each error bar represents the standard error of mean calculated from at least 15 data points. **e** Optical absorbance of different SWBM samples (left y-axis) and AM1.5 G (magenta curve) solar irradiance (right y-axis)

Our SWBM samples are produced through single-step and scalable femtosecond (fs) laser processing of thin aluminum (200 μm) foils (see methods; Supplementary Note 2)^40–45^. The SWBM surfaces consist of an array of parallel micro-scale grooves and ridges (see Fig. 2a; Supplementary Note 3). The micro-grooves and micro-ridges are superimposed by a range of nanostructures. By controlling laser processing power, we can produce SWBM samples with deeper and wider microgrooves at a higher laser power but with shallower and narrower microgrooves at a lower laser power. Fig. 2b shows the depth profile of the four SWBM samples with around 80 μm, 100 μm, 120 μm, and 150 μm, respectively by using different laser power (0.6, 0.9, 1.2, and 1.5 W). These samples are labeled as SWBM-0.6, SWBM-0.9, SWBM-1.2, and SWBM-1.5 hereafter.

Water transport rate in a micro-capillary is determined by capillary pressure, gravity, water inertia, and viscous pressure^46^. However, considering the small amount of water in the capillary, gravity and inertia can be neglected. Therefore, the Washburn-Rideal equation provides a rather accurate estimation of the water wicking dynamic in this case^47^:

1 $${W}_{{front}}^{2}=K\left(\alpha ,\theta \right)\frac{\gamma h}{\mu }t$$

where W_front_ is wet front position at a time t. Constants $\gamma$ and $\mu$ are surface tension and dynamic viscosity of the fluid, respectively, h is depth of the groove, and $K(\alpha ,\theta )$ is a geometric term that depends on the groove angle ${\rm{\alpha }}$ and liquid/solid contact angle ${\rm{\theta }}$ (Fig. S5). For an experimental validation, we mount SWBM samples vertically on a crane structure, which is positioned on a z-stage. Subsequently, we decrease the height of the sample until the bottom of the sample just touches the reservoir water surface (see methods; Supplementary Note 4). As soon as the SWBM touches the water surface, water runs uphill against gravity on the sample surface at an unprecedented high speed. The water wicking dynamics is video recorded using a high-speed camera. Snapshots at different times are shown in Fig. 2c and Fig. S6d, where W_front_ are marked with white lines. We can see that the water wet front position advances proportional to the square root of time for all samples, which agrees well with the theoretical prediction (Eq. 1). For the four studied samples, the wicking speed increases with the laser power (Fig. S6c). SWBM-1.5 has the strongest wicking effect, on which water runs uphill with an initial velocity of 8 cm/s and an average velocity of 2 cm/s (Fig. S6c). The amount of water supply due to capillary effect can then be estimated using the average water wet front velocity and the cross-section area of the microcapillary (Fig. S7). Fig. 2d shows the amount of water wicked onto different samples. SWBM-1.5 has the highest water supply rate due to its strongest wicking effect and the largest capillary cross section area.

Besides the superwicking effect for water transport, SWBM also has a nearly ideal absorption for sunlight. We have shown that light trapping in the microgrooves and hybridization of multiple surface plasmon resonance modes from the micro and /nanostructures enable near perfect optical absorption over a broad spectral range^18,48–51^ (Fig. 2e). The average solar absorbance is given by $\bar{\alpha }={\int }_{0}^{\infty }\alpha (\lambda )I(\lambda )d\lambda /{I}_{{total}}$; where ${I}_{{total}}$ is total solar irradiance and I(λ) is solar irradiance at a given wavelength. At 1.2 W laser processing, we obtain an optimal solar absorption for SWBM with ∼ 98% absorbance at the peak solar irradiance while ∼ 92% average absorbance in the entire solar region. SWBM-1.2 has the highest broadband optical absorption because it has the optimum combination of microstructures and nanostructures over a range of sizes (see Supplementary Note 5)^52^.

### Solar thermal interfacial desalination of actual ocean water

**Fig. 3:**
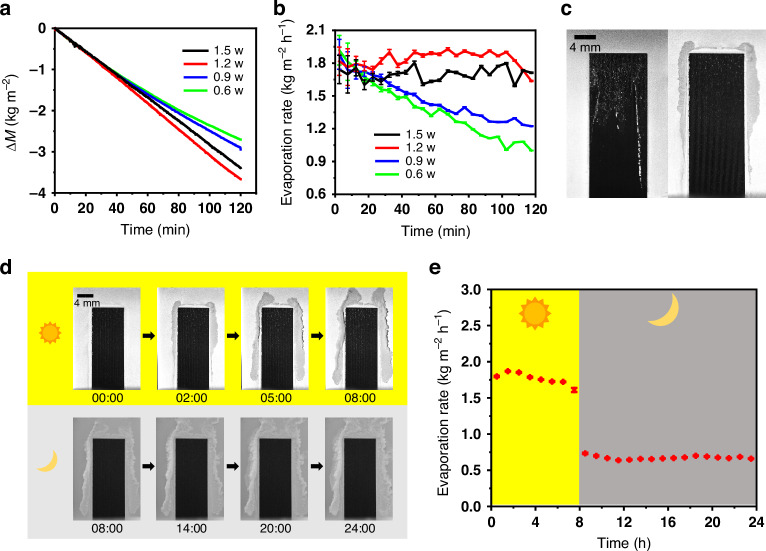
SWBM surface morphology optimization for self-cleaning. **a** Evaporation mass flux for SWBM samples fabricated using different laser power and (**b**) corresponding evaporation rate under one-sun illumination. Each error bar represents the standard error of mean calculated from at least 60 data points. **c** Surface photographs of two different SWBM samples after 2 h of evaporation; (left) sample SWBM-0.6 showing salt crystallization and accumulation on the active region and (right) sample SWBM-1.2 demonstrating efficient self-cleaning. **d** Time lapse images of SWBM-1.2 surface showing self-cleaning dynamics when operating (top panel) under one solar illumination and (bottom panel) dark condition. **e** 24 h evaporation rate of the SWBM-1.2 sample. Each error bar represents the standard error of mean calculated from at least 60 data points

After wettability and optical absorbance measurements, we test solar-thermal desalination performance for each SWBM sample for evaporating actual ocean water under one sun irradiation for a period of two-hour. In this study, unless otherwise stated, actual ocean water is Atlantic Ocean water obtained around Fire Island, New York, USA. For all indoor experiments, the room temperature is controlled around 22.5° C and relative humidity is controlled around 35%. The SWBM evaporator is suspended freely using a rigid and unextendible hook from the crane structure (See methods, Fig. S10). The water reservoir is placed onto a digital weighing balance to measure the water mass over time. The lower end of the SWBM touches the water surface through an adjustable rectangular slit where the slit width is minimized to avoid direct evaporation from the reservoir. The SWBM surface is normally illuminated with a spatially uniform (1 kW/m^2^) and collimated light beam from a solar simulator having an AM1.5 G filter. The dependence of evaporation mass flux and corresponding evaporation rate on time for different SWBM samples are shown in Fig. 3a, b. The SWBM-0.6 and SWBM-0.9 samples have marginally higher initial evaporation rates (Fig. 3b) due to a better heat localization enabled by their lower water supply rate (Fig. 2d). However, at higher evaporation rates, the relatively low water supply rate for these samples leads to salt crystallization and capillary blockage on the evaporating surface. As shown for the left sample (SWBM-0.6) in Fig. 3c, the top half of the evaporator’s surface is entirely covered by crystallized salt resulting in an over 45% drop from its initial evaporation rate within two hours. In contrast, the evaporator surfaces for SWBM-1.2 and SWBM-1.5 remain clean throughout the period, resulting in stable and high average evaporation rates of ∼ 1.84 ± 0.01 and ∼ 1.70 ± 0.01 kg m^−2^ h^−1^, respectively. As will be discussed later, for SWBM-1.2 and SWBM-1.5 samples, the salt crystallization initiates from the edge and expands outward subsequently, thus avoiding salt accumulation in the active area. (see Fig. 3c right; Supplementary Note 6). Unlike SWBM-0.6 and SWBM-0.9, the evaporation rate for SWBM-1.2 and SWBM-1.5 after 1 h of operation is even slightly larger than the initial values, indicating that the salt crystals accumulating at the passive regions of the evaporator are contributing to the evaporation. The SWBM-1.2 sample outperforms the SWBM-1.5 due to a combined effect of a larger optical absorbance (see Supplementary Note 5), a better heat localization (because of a lower water transport rate in a shallower groove), and a larger air-water interface length (see Supplementary Note 7). Since SWBM-1.2 has the highest ocean water evaporation rate, we will focus on this sample for the rest of the discussion.

To test our optimized ABF-STIC, we run the device continuously over the 24-hour cycle, including 8-hour 1-sun solar irradiation and 16-hour dark time imitating the actual solar cycle. The ABF-STIC’s surface is imaged throughout the operation to record salt-crystallization dynamics (see Supplementary video SV1). The top-panel of Fig. 3d shows time-lapse photographs of the ABF-STIC surface during day hours while images in the lower panel show the surface during night hours. The first image shows the initial moment when the SWBM’s bottom just touches water. The active region wicks the water uphill and spread it to the surrounding passive region. As time goes on, salt will start to crystallize at the edges of water boundary in the passive region and grow outwards. The salt crystalizes almost symmetrically on each side of the passive region. However, salt accumulates faster on the sides than at the top and we hypothesis that it is most likely due to the spatial variation in water transport rate because of the nature of the laminar water flow (see water boundary in Fig. 2c and Supplementary Note 8). Fig. 3e shows the corresponding evaporation rate over the 24 h period. The average evaporation rates are 1.76 ± 0.03 and 0.67 ± 0.01 kg m^−2^ h^−1^ during day and night hours, respectively. We obtain a solar to vapor conversion efficiency of ∼ 74% in evaporating actual ocean water (see Supplementary Note 9).

### Ocean water salt harvesting without brine discharge

**Fig. 4:**
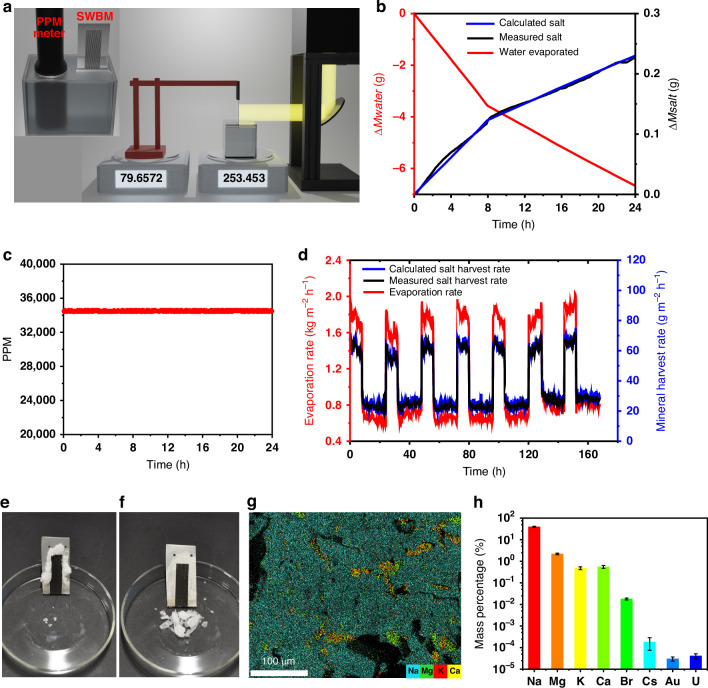
Demonstrating ABF-STIC performance and long-term stability in desalinating actual ocean water and salt harvesting with zero liquid discharge. **a** Schematic diagram of the designed 2-balance operando experimental platform. The inset shows the special water reservoir integrating a PPM reader for in-situ measurements of the reservoir water salinity over time. **b** The amount of water evaporated (left y; red line), salt harvested (right y; black line), and calculated salt harvesting (right y; blue line) when treating Atlantic Ocean water collected from Fire Island, NY, USA and (**c**) corresponding PPM of the ocean water reservoir during the experiment. **d** Rate of evaporation (left y; red line), measured salt harvesting rate (right y; black line), and calculated salt harvesting (right y; blue line) rate from the ocean water for 7 consecutive days. Photographs of the ABF-STIC device (**e**) with accumulated salt and (**f**) after removing the accumulated salt. The two holes on the SWBM are drilled for mounting. (**g**) Elemental mapping of the collected salts; sodium, magnesium, potassium, and calcium and (**h**) the corresponding mass percentage with cesium, gold, bromine, and uranium

In conventional salt-rejecting interfacial evaporators, salt accumulates at the evaporator surface under solar radiation and self-dissolves back to the water reservoir at nighttime when the evaporation rate is lower. This leads to an increased salinity in the reservoir over time and ultimately leads to an untreatable brine discharge. In contrast, the collected salts are confined in our ABF-STIC passive regions throughout the day and will not dissolve back to the reservoir to increase its salinity. To demonstrate this, we design and build an operando experimental platform that simultaneously and continuously measures water evaporation mass, salt harvesting mass, and reservoir water salinity (see Supplementary Note 10). The operando platform (Fig. 4a) includes two balances, a miniature crane structure, and a 3D printed water reservoir (Fig. S16b) that can integrate a digital ppm reader. Like the previous setup (Fig. S10), the SWBM evaporator is suspended freely using a rigid and unextendible hook from the crane structure secured to one of the balances (Fig. 4a). The SWBM lower end passes through the slit and touches the water in the reservoir that is placed on the second balance. Water evaporation leaves salt on the passive region of the evaporator and therefore, the mass increase on the first balance (left one in Fig. 4a) characterizes the amount of salt harvested, while the mass decrease on the second balance (right one in Fig. 4a) reflects the loss of water and minerals. The reservoir salinity is monitored in-situ throughout the experiment using the integrated ppm reader. The experiment is first performed for a period of 24 h including 8 h under solar irradiation and 16 h of non-solar operation. For long-term durability and stability demonstration, the cycle is repeated for 7 consecutive days.

Figure 4b shows mass of the evaporated water (left; redline) and mass of the collected salt (right; black line) over time. As discussed before, the system has stable performance due to self-cleaning functionality of the evaporator; therefore, the water mass loss is linear, but the slope varies between daytime and nighttime. The average rate of water evaporation is 1.79 ± 0.03 and 0.77 ± 0.01 kg m^−2^ h^−1^ during the solar and non-solar periods, respectively. As expected, the time variation in the amount of collected salt follows the trend of time variation in the water evaporation. The average rate of salt harvesting is 61.28 ± 2.45 and 25.83 ± 0.52 g m^−2^ h^−1^ during the solar and non-solar periods, respectively. To demonstrate that nearly all the salt present in the evaporated water is collected by the ABF-STIC surface, we calculate the amount of salt (right; blue line) present in the evaporated water using expression: ${M}_{S}=\rho \times {M}_{w}\times {10}^{-6}$, where M_s_ is the mass of salt present in the evaporated water, M_w_ is the mass of the evaporated water, and $\rho$ is the salinity of the water in PPM recorded by a PPM meter throughout the experiment. We observe that the amount of collected salt is very close to the amount of salt that is present in the evaporated water. This finding implies that almost 100% of the salt is collected by the ABF-STIC surface and thus the accumulated salt does not dissolve back into the reservoir. This result is further supported by the PPM measurements of the reservoir water salinity over time (Fig. 4c), which is constant throughout the 24 h measurement period. Over a 24 h cycle, about 10% reservoir water evaporates. If the accumulated salt gets dissolved back to the reservoir, the salinity should increase over time rather than remaining the same. Therefore, our ABF-STIC does not discharge brines. To demonstrate the durability of the system, we run the experiment continuously for a week. Fig. 4d shows water evaporation rate (red curve; left), measured salt harvesting rate (black curve; right), and calculated salt harvesting rate (blue curve; right) during the entire 7-day test period. The results show that the water evaporation and salt harvesting rates are stable throughout the test period, and almost 100% of the salt contained in the evaporated water is harnessed. The average evaporation and salt harvesting rates for the 7-day test period are 1.76 ± 0.04 kg m^−2^ h^−1^ and 61.74 ± 2.46 g m^−2^ h^−1^ during daytime and 0.70 ± 0.01 kg m^−2^ h^−1^ and 24.66 ± 0.49 g m^−2^ h^−1^ at night, respectively. The salt accumulated on the passive regions of ABF-STIC can be easily harvested by simple scratching without touching the active regions (Fig. 4e, f). Additionally, the SWBM maintains its optical and wetting functionalities after seven consecutive days of evaporation (see Supplementary Note 11). Elemental composition of the collected salt is obtained using energy dispersive X-ray absorption (EDAX) spectroscopy and mapping. Sodium is the most abundant, about 40% in weight, in the collected salt followed by 2.2% magnesium, 0.48% potassium, and 0.55% calcium (Fig. 4g). In addition to commonly known salts (Na, K, Ca, and Mg) found in ocean water, ABF-STIC also harvests economically valuable salts, including gold, cesium, bromine, and uranium (Fig. 4h). The harvested salt by ABF-STIC without further separation/purification may find limited use. Addressing this aspect involves careful material design and process optimization, which can be the focus of future study. For a preliminary example, we functionalized the ABF-STIC microcapillary surface by depositing metatitanic acid (HTO) nanoparticles, which readily and selectively trap lithium ions on the active area while leaving other salts in the passive area during desalination (see Supplementary Note 12 for details)^53–55^.

## Discussion

### Mechanism of self-salt rejection and salt harvesting

**Fig. 5:**
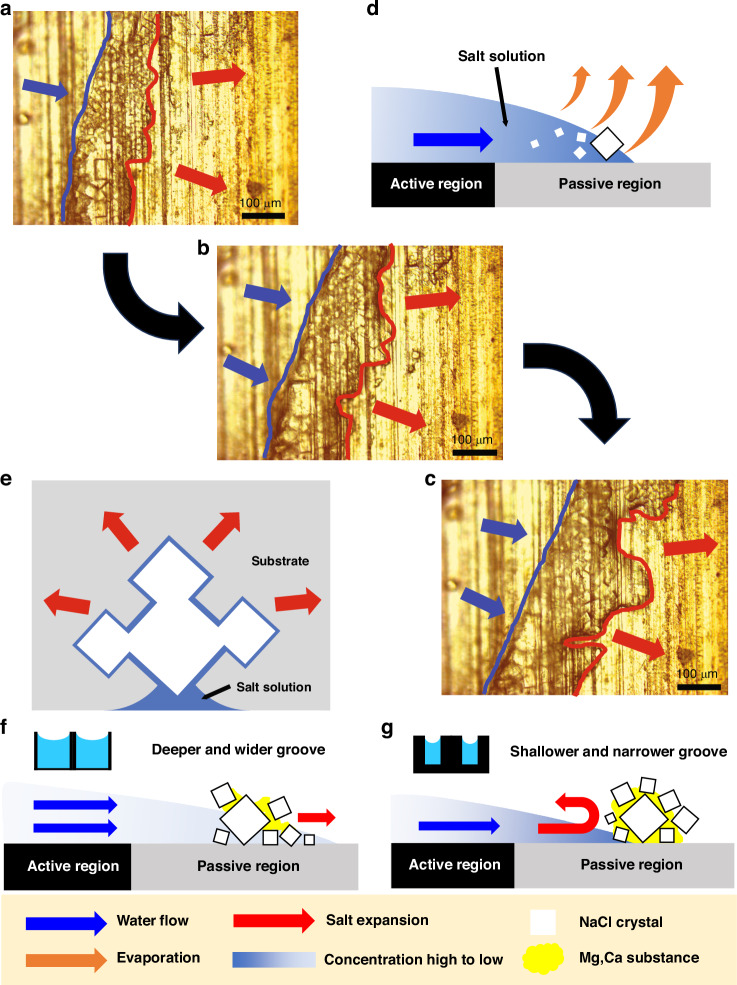
Mechanism of salt-crystallization away from the active regions of the ABF-STIC surface. **a**–**c** Time-lapse microscope view of the salt growth phenomenon recorded over a 10-minute duration. The blue lines mark the saltwater boundary, and the red lines mark the salt boundary. The time interval between each image is 5 min. **d** Schematic (side view) showing evaporation induced edgeward flow of saltwater and salt nucleation at the edge. A thicker and longer arrow shows relatively higher evaporation near the solid-liquid-air contact line. **e** Schematic (top view) of self-amplified salt-creeping process. **f** Schematic showing active region with deeper and wider grooves has stronger dissolving capillary flow for self-cleaning. **g** Schematic showing active region with shallower and narrower grooves has weak dissolving capillary flow resulting in clogging

To understand the mechanisms of how the SWBM surface self-rejects salt and harness nearly 100% of salt with zero liquid discharge, we examine the salt crrystallization process using a microscope (see Supplementary Note 13). Figure 5a–c illustrates a time lapse of the magnified view of the salt crystallization process by focusing on a local region near the saltwater boundary of SWBM-1.2. Initially (Fig. 5a), salt crystallizes at the saltwater boundary just outside of the active region (marked by blue line), then advances forward forming a separate salt boundary in the passive region (marked by red line). As time goes on, both the saltwater boundary (blue line) and the salt boundary (red line) expand outward (Fig. 5b, c). As a result, no salt crystal is observed in the active region. By contrast, inward salt expansion is observed for SWBM-0.6 (Fig. S23b).

The observed salt growth can be explained by two sequential physics mechanisms: the coffee ring effect followed by salt creeping^56–59^. The coffee ring effect comes from evaporation-induced capillary flow^56^. For a drop of solution on a substrate, the evaporation rate is the highest at the three-phase lines, where solution, substrate, and air meet. To compensate for the evaporation loss, the solution flows towards the edge carrying with the solutes. As a result, the solution at the edge will first reach saturation concentration and consequently induce crystallization at the boundary. For the SWBM, salt water is continuously supplied from the reservoir into the micro capillaries. At the boundary between the active and passive regions, water will spill over the active regions towards the passive regions (Fig. 5d). Therefore, a saltwater boundary (blue line in Fig. 5a–c) will form on the surrounding passive area. When evaporation happens, the solution concentration gradually increases. Then, the subsequent refilling flow will push this high-concentration solution from the micro capillaries towards the saltwater boundary. As time goes on, the solution concentration on the edge becomes the highest and will reach saturation first, resulting in crystallization. To verify the coffee ring effect, we have performed both theoretical simulations and experiments (see Supplementary Note 14). Both the simulation and experimental results show that only the solution at the saltwater boundary on the passive region reaches saturation concentration, which agrees with our observation that salt crystallization only happens on the passive regions of the evaporator.

After crystallization, salt creeping comes into play as the salt crystals start expanding outward. Salt creeping is a phenomenon where salt crystal forming ahead of the evaporating saltwater boundary^60^. This phenomenon is induced by the thin film solution wicking on the surface of the salt crystal (Fig. S23b, c). At the water–salt crystal interface, the thin water film dissolves the nearby salt. This solution is then transported through the porous salt structure by capillary action and subsequently re-crystallizes at the outer surface, driving outward salt expansion^58^. This is a self-amplifying effect because these newly formed salt crystals will continuously wick saltwater, dissolve the region marked by the blue line, and stimulate further crystallization (Fig. 5e)^59^. Thus, both the saltwater and salt boundaries will continuously expand outward. Therefore, it appears that our SWBM self-rejects salt. We note that salt creeping is affected by the porosity of the salt crystals. Sodium chloride, mostly constituting sea salt, is porous and easily wicks water, which makes salt creeping easy. However, the magnesium sulfate present in ocean water may obstruct the pores among the sodium chloride crystals and impedes the water transport through these pores^34^ and reduces the salt creeping. This explains why capillary clogging is a common issue in most water transport systems. To mitigate capillary clogging, a sufficiently strong dissolving capillary flow must reach the salt boundary to break open the sodium chloride crystals so that the water film can continuously flow outward. The effectiveness of this dissolving flow depends mainly on the solute concentration of the flow. SWBMs with both deeper and wider grooves enhance the strength of the dissolving flow. The larger cross-sectional area associated with deep and wide grooves increases the water volume in the microcapillary, maintaining a lower solute concentration during evaporation. This lower concentration not only drives a greater concentration gradient for dissolution but also prolongs the available dissolution time. As a result, the flow dissolves the salt crystals, penetrates through the crystal matrix, and promotes outward salt boundary expansion (Fig. 5f). In contrast, SWBMs with shallower and narrower grooves yield weaker capillary flows that tend to crystallize prematurely, leading to backward crystallization and eventual clogging (Fig. 5g). Figure S26e summarizes the crystallization behaviors for various SWBM samples when treating real ocean water under one sun illumination. We identified a clear boundary (marked in red in Fig. S26e) distinguishing different crystallization behaviors: SWBMs with deeper and wider grooves tend to exhibit self-cleaning, while those with shallower and narrower grooves are more prone to clogging. For example, the SWBM in a previous work (SWBM-S1: reproduced in Supplementary Note 15)^38^ has a large groove depth (140 μm) but a narrow groove width (30 μm); the relatively small cross-sectional area leads to a higher solute concentration at the salt boundary, causing inward salt expansion and eventual capillary clogging. In contrast, SWBM with stronger dissolving capillary flow, such as SWBM-1.2 and SWBM-1.5, dissolve the salt crystals, keeping the wicking channels unblocked and ensuring the active area remain clean during ocean water desalination. Overall, SWBMs with groove depth > 110 µm and width > 50 µm allow self-cleaning when treating real ocean water.

### Solar trackable desalination and test across multiple ocean waters

**Fig. 6:**
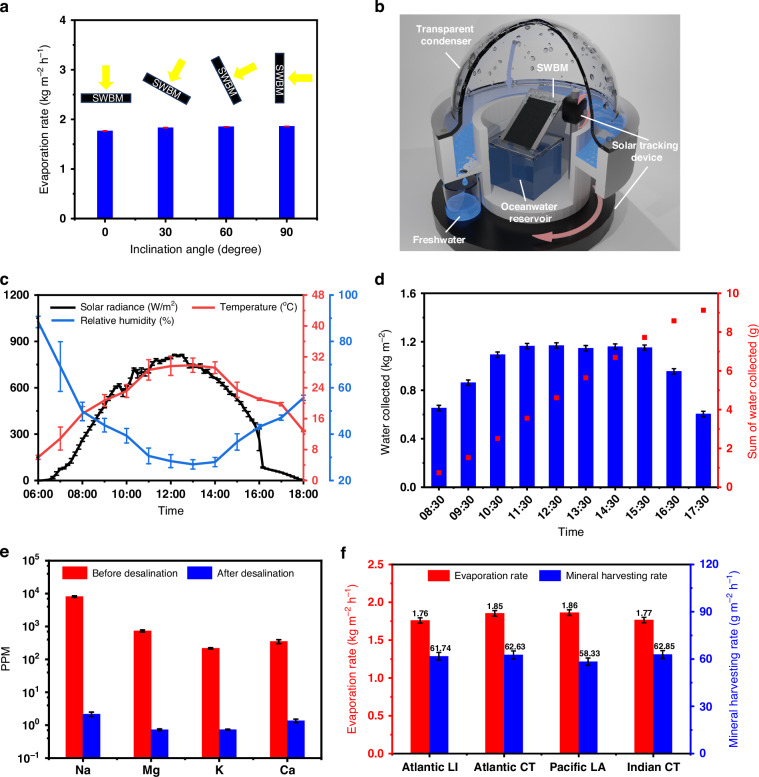
Practical demonstration in solar tracking and treating several actual ocean water samples. **a** SWBM evaporation performance mounted at different inclination angles. Each error bar represents the standard error of mean calculated from at least 60 data points. **b** Schematic of ABF-STIC integrated with a solar trackable condensation system. **c** Measured solar radiance (left y-axis; black line), ambient temperature (right y-axis; red line), and relative humidity (right y-axis; blue line) during the experiments. Each error bar represents the standard error of mean calculated from at least 60 data points. **d** Freshwater production rate (left y-axis; histogram) and sum of the produced fresh water (right y-axis; scattered data) during the outdoor experiment. Each error bar represents the standard error of mean calculated from at least 15 data points. **e** Salt concentration of Atlantic Ocean water samples before (red bar) and after (blue bar) the solar-thermal desalination. Each error bar represents the standard error of mean calculated from at least 15 data points. **f** Water evaporation (left y-axis; red bar) and salt harvesting (right y-axis; blue bar) rates for different ocean water samples collected from Atlantic Ocean (Fire Island, USA), Atlantic and Indian Ocean (Cape L’Agulhas, South Africa), and Pacific Ocean (Los Angeles, USA). Each error bar represents the standard error of mean calculated from at least 15 data points

A major advantage of ABF-STIC is solar trackability since the 2D wicking surface can pull water uphill against gravity, allowing us to position the surface in any orientation. To demonstrate ABF-STIC’s solar trackability, we first examined the evaporation performance of SWBM when it is mounted at different inclination angles. When the inclination angle decreases, the evaporation rate only drops slightly while self-cleaning effect is virtually unchanged (Fig. 6a, Supplementary Note 16). Therefore, ABF-STIC can track the solar motion to maximize solar flux and desalination performance with little efficiency loss throughout the day. Afterwards, we built a solar-tracking ocean water desalination system by integrating ABF-STIC with a transparent hemispherical dome (Fig. 6b). In outdoor measurements, sunlight is incident onto the ABF-STIC surface through the transparent dome to generate water vapor, which is condensed on the interior hydrophobic surface of the dome and collected by the sliding walls. We carried out an outdoor experiment on March 04, 2024, on the roof of the Wilmot Building at University of Rochester, NY, USA (43.16° N, 77.61° W) (see Supplementary Note 17). We measured the Atlantic Ocean water (Fire Island, USA) desalination rate throughout the day using a scaled 3 cm × 3 cm SWBM (see Supplementary Note 18), from 8:30 a.m. to 5:30 p.m. For every hour during the day, the azimuth and inclination angles of the evaporator is adjusted to face the Sun to capture the maximum solar radiation. During the experiment, the solar radiation, outdoor temperature, and the relative humidity were recorded (Fig. 6c) and the corresponding rates of desalination were plotted in Fig. 6d. The highest desalination rate occured at 12:30 h, reaching 1.169 kg m^−2^ h^−1^ when the sun is the most intense (around 0.8 sun). Therefore, the 1-sun normalized water production rate is 1.461 kg m^−2^ h^−1^. We collected a total of 9.3 g freshwater along with 0.343 g of sea salt from the ABF-STIC with a 9 cm^2^ surface area over the course of 9 hours. This is equivalent to generating 10.33 liters m^−2^ of freshwater and 0.38 kg m^−2^ of sea salt per day. The salinity of the desalinated water is found well below the WHO and EPA standards for safe drinking water (Fig. 6e). Besides the Atlantic Ocean water used in our study, we further tested three other types of ocean water collected from a wide variety of geographical locations, including both Atlantic and Indian Ocean waters around Cape L’Agulhas, South Africa and Pacific Ocean water around Los Angeles, USA to test the potential variability in ocean composition globally. Fig. 6f shows water evaporation (left y-axis; red bar) and experimentally measured (right y-axis; blue bar) salt harvesting rates for the four ocean water samples. It turns out that evaporation rates and salt harvesting rates are almost the same for all the four ocean water samples and the desalinated water from these oceans can all be used for drinking (see Supplementary Note 19).

## Conclusions and outlook

In this work, we developed a true sustainable and environmental-friendly solution for desalination and salt extraction of actual ocean water. Besides desalination, we envision that ABF-STIC can also be used to for a range of inorganic and organic solutions for solute separation and solvent recovery. Additionally, the active area of ABF-STIC could be functionalized with linker molecules to selectively collect desirable salts from ocean water or certain solutes from a solution.

## Materials and Method

### Femtosecond laser fabrication of SWBM samples

The **e**xperimental setup for fabricating the SWBM samples is shown in Fig. S1a and described in our previous work^38,40^. In a typical experimental procedure, a burst of femtosecond laser pulses from a Ti: Sapphire laser (Astrella, Coherent), operating at 800 nm wavelength. 35 fs pulse width, and 1 kHz repetition rate, is focused on the surface of a 200 µm thick aluminum foil, mounted on a x-y translational stage, at a normal incidence. The substrate is raster scanned with the interline spacing of 100 µm. For fabricating different SWBM samples, a range of laser pulse energies at 600 µJ, 800 µJ, 1.2 mJ, and 1.5 mJ are used.

### Hemispherical optical absorbance of SWBM samples

The SWBM’s hemispherical optical reflectance is measured in the spectral range of 0.25–2.5 μm using a PerkinElmer Lambda-900 double-beam spectrophotometer coupled with a 50 mm-diameter integrating sphere. Since the SWBM is opaque, the absorbance can be calculated using the measurement of reflectance with the equation: A = 1–R.

### SWBM Surface profile and surface morphology measurements

A 3D scanning laser microscope (Keyence VK 9710-K) is used to measure the surface/depth profile of the hierarchical microstructures on the SWBM surface, with a height resolution of 500 nm. Additionally, surface morphology of the samples is measured using a Zeiss-Auriga scanning electron microscope.

### Wetting dynamics measurements

As fabricated SWBM sample is vertically mounted on a crane structure, positioned on a z-stage. Subsequently, we decrease the height of the sample until the bottom of the sample just touches the reservoir water surface. As soon as the SWBM touches the water surface, water runs uphill against gravity on the sample surface. The wetting dynamic is recorded with a high-speed camera.

### Calibrating the solar simulator and power on the sample plane

The solar simulator (Sanyu) with an AM1.5 G airmass filter is first calibrated for 1 Sun (1,000 W m^−2^) using a NREL-certified PV reference solar cell (PV Measurements). The solar simulator generates a vertically collimated square beam (10 cm × 10 cm), which is then reflected horizontally using a 45° mounted plane mirror. A shutter is implemented in the beam path so that the beam shape matches the active region of the sample. We use a thermopile power meter (FieldMax II TO, Coherent), set at a wavelength of 500 nm to calibrate for 1 sun concentration. Since the head of the pyroelectric power meter is circular in shape with a diameter of 19 mm (area, 2.83 cm^2^), 1 sun concentration is acquired when the reading becomes 283 mW.

### Indoor water-evaporation and salt harvesting measurements

Water-evaporation and salt harvesting rates are measured using a two-balance setup. The details of the setup are described in the manuscript and in Supplementary note 8. For all indoor experiments, the room temperature is controlled around 22.5 °C and humidity is controlled around 35%. The sea water sample was collected from Atlantic Ocean, Fire Island, USA. The laboratory temperature and humidity are maintained throughout the experiments. First, the system without the SWBM in dark is measured for 1 h for reference. This will give the systematic error due to natural evaporation loss. Then, the SWBM sample is integrated for the measurement. For both balances (Radwag AS 60/220.R2 and Radwag PS 360.R2), the mass reading is recorded every 10 s. Several rounds of measurements are taken, and the most representative data is presented in the manuscript.

### Reservoir water salinity measurements

A PPM meter (PCE-PHD 1, PCE instruments) is incorporated with a 3D printed water reservoir to measure the PPM of reservoir water constantly and simultaneously with the water evaporation and salt harvesting data. The dimension of the circular hole is designed to be the same as the size of the PPM meter to minimize natural evaporation loss. The PPM meter is connected to a PC so that its reading can be recorded every 10 s.

### Compositional mapping of the collected salt salts

Energy dispersive X-ray absorption spectrometer (AMETEK EDAX system) attached with Zeiss-Auriga scanning electron microscope is used to map distribution of different salts in the harvested salt.

### Outdoor water-evaporation measurements

The outdoor experiment setup is shown in Fig. 6b. Since we need to do solar tracking, we can’t use two-balance setup anymore. Therefore, only water-evaporation rate is recorded using Radwag PS 360.R2. Again, the mass reading is recorded every 10 s. Windshields are installed around the setup to avoid the wind effect on the evaporation. Moreover, we used a one-face-open cubical box to cover the sample on the balance so that the measurement is in stable condition. During the experiment, solar radiation (Apogee 420 pyranometer), ambient air temperature, and relative humidity (Sensirion SEK-SHT35) are also recorded every 5 s. Every one hour, we adjust the position of SWBM so that it always faces sunlight vertically.

## Supplementary information

Supplementary Information for: Additive-free and brine-discharge-free solar-thermal desalination with simultaneous complete mineral mining from ocean water

Self-cleaning video

## Data Availability

The data that support the findings of this study are available from the corresponding author C.G. on reasonable request.
