# Supplementary material for: Additive-free and brine-discharge-free solar-thermal desalination with simultaneous complete mineral mining from ocean water
Source: Light Sci Appl. 2026 May 27;15:246. doi: 10.1038/s41377-026-02315-4 (PMC13212987; doi:10.1038/s41377-026-02315-4)
Supplement: Supplementary file 1 — Supplementary Information for: Additive-free and brine-discharge-free solar-thermal desalination with simultaneous complete mineral mining from ocean water [file 41377_2026_2315_MOESM1_ESM.docx]

**Supplementary information for:**

Additive-free and brine-discharge-free solar-thermal desalination with simultaneous complete mineral mining from ocean water

Luheng Tang, Subhash C. Singh*, Ran Wei, Tianshu Xu, and Chunlei Guo*

The Institute of Optics, University of Rochester, Rochester, NY-14627, USA

*Corresponding author: [ssingh49@ur.rochester.edu](mailto:ssingh49@ur.rochester.edu), [guo@optics.rochester.edu](mailto:guo@optics.rochester.edu)

**Supplementary Note 1**

**Salt accumulation on an open capillary surface that is not designed for self-cleaning**

A control experiment is performed to compare the long-term evaporation performance of an open capillary surface that is not designed for self-cleaning and a porous fiber coated with black paint (as shown in **Fig. 1d**) under solar radiation. For the open capillary surface, the surface was covered by salt crystals even when treating simulated ocean water within 2 hours and flushing is needed to remove the accumulated salt for later-on usage (**Fig S1a-d**). For the porous material surface, the evaporation rate is recorded when treating real oceanwater under 1 sun illumination. The surface was heavily covered by salt crystals due to capillary clogging, leading to a significant drop in evaporation rate (**Fig S1e-f**).

**
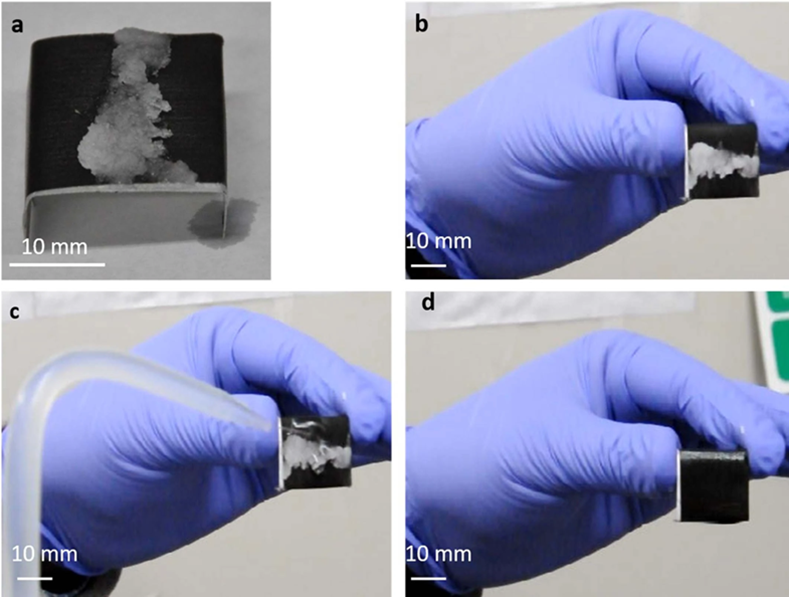
**

**
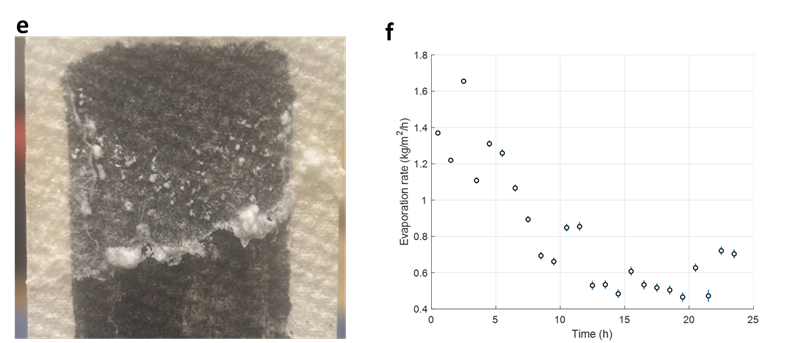
**

**Figure S1 (**a-d reproduced from Singh et al., Nat Sustain (2020), licensed under CC BY 4.0^1^**,** the figure is original to the cited paper and does not contain third-party material requiring separate permission**.):** Accumulation of salt on the active area of an unoptimized open capillary surface. (a, b) the surface shows excessive salt accumulation after two hours of evaporation of simulated seawater with concentrated (104 mg/liter) NaCl solution in the horizontal plane. This surface requires regular maintenance as it can only transport and evaporate water before the surface is covered by salt. (c) the accumulated salt is removed by water flushing for reuse, (d) After cleaning, the surface can be reused. e) Accumulation of salt on the active area of porous material, f) the evaporation rate of the porous material under 1 sun illumination during 24 hour period, the rate drops significantly due to clogging.

**Supplementary Note 2**

**Superwicking Black Metal (SWBM) surface fabrication and characterizations**

Experimental setup for the fabrication of the SWBM surface is described in **Fig. S2a**. The femtosecond laser beam (Ti: Sapphire, Coherent Astrella) operates at 800 nm wavelength, and 1 kHz repletion rate. The 200 µm thickness aluminum foil is mounted on an XY translation stage and the laser beam is focused on the target surface using a planoconvex lens and scans line-by-line with the interline spacing of 100 µm.


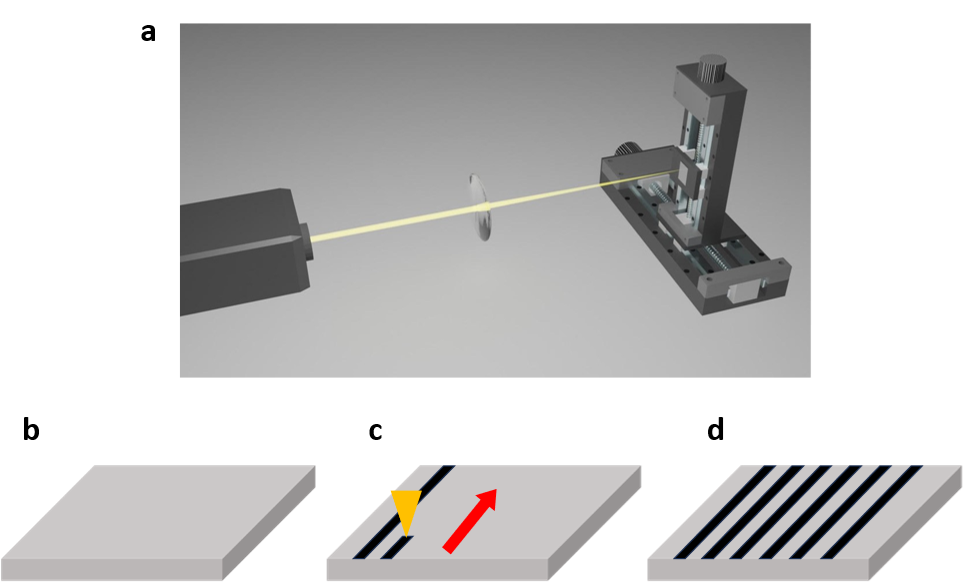


**Figure S2**: (a) Schematic of laser fabrication of the SWBM. (b-d) Schematic of laser processing: b) unprocessed sample, c) processing, d) processed. The yellow arrow represents laser and red arrow represents the direction of laser processing, the grey color cubic represents passive aluminum foil and black stripe represents the laser treated region.

**Supplementary Note 3**

**Superwicking Black Metal surface morphology and surface topography**

**i) Surface morphological measurements using SEM**

**
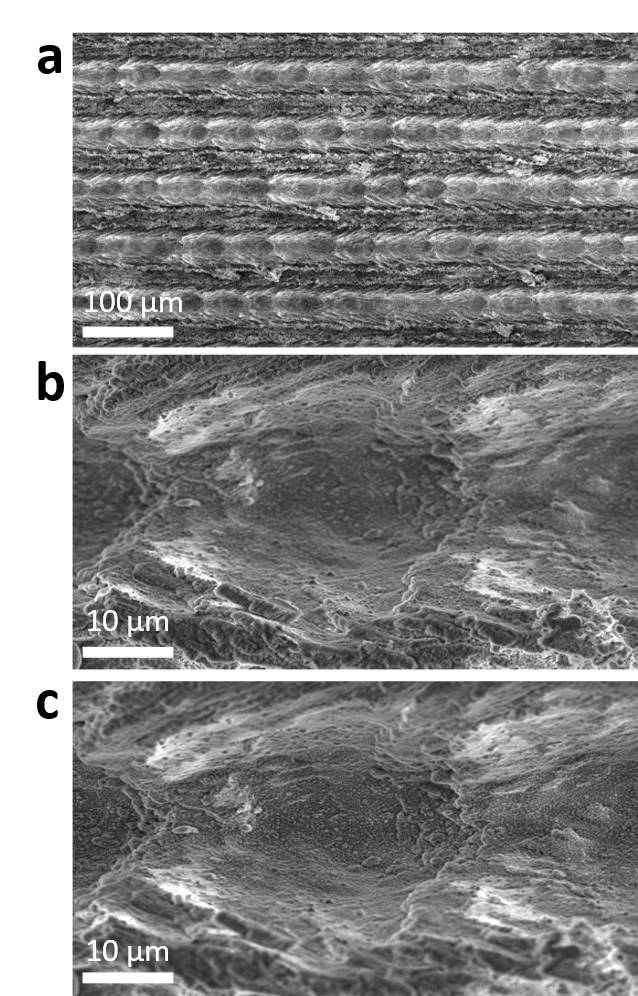
**

**Figure S3**: (a) SEM image for micro/nanostructures on the SWBM sample together with high magnification image of (b) micro-ridge and (c) micro-groove on the SWBM surface.

**ii) Surface topography measurements using 3D laser confocal microscope**

**
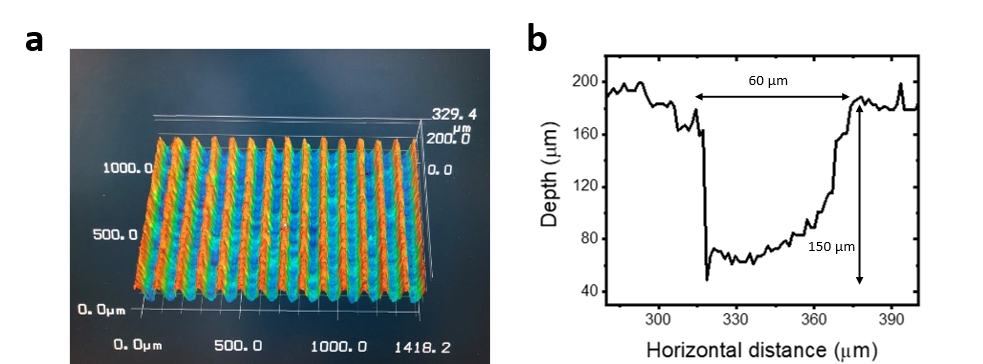
**

**Figure S4**: a) 3D surface topography of SWBM-1.2. b) An enlarge view of one representative micro-capillary of SWBM-1.5. The width of the micro-capillary is approximately 60 μm.

**Contact angle measurement of Superwicking Black Metal**

**
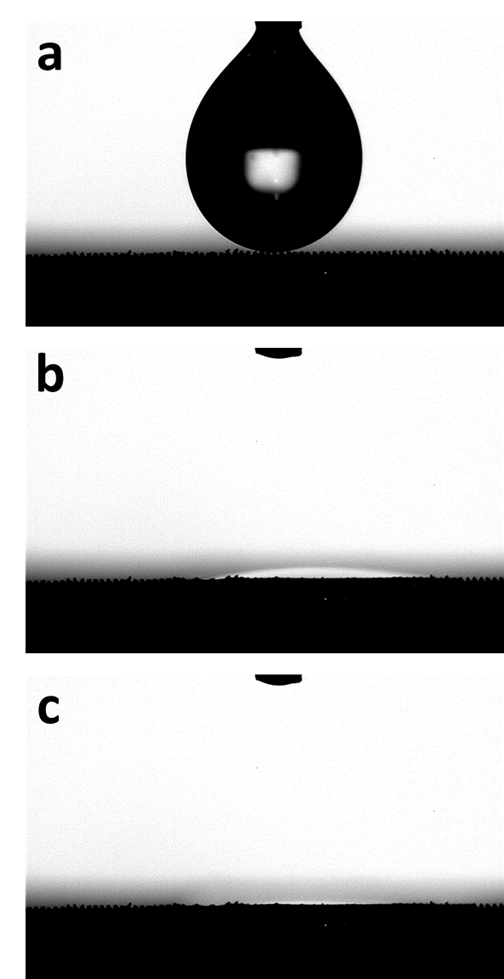
**

**Figure S5**: Snap shots of water droplet touching the SWBM surface. The time step between each photo is 0.2s. The contact angle of the SWBM is 1.732 degree. In the first image, the surface appears rough due to the presence of micro-capillaries. When a water droplet contacts the SWBM, it spreads out extremely quickly. In the last image, we observe that the surface becomes flattened because the water droplet fills the micro-capillaries.

**Supplementary Note 4**

**Experimental measurement of wicking dynamics of SWBM samples**


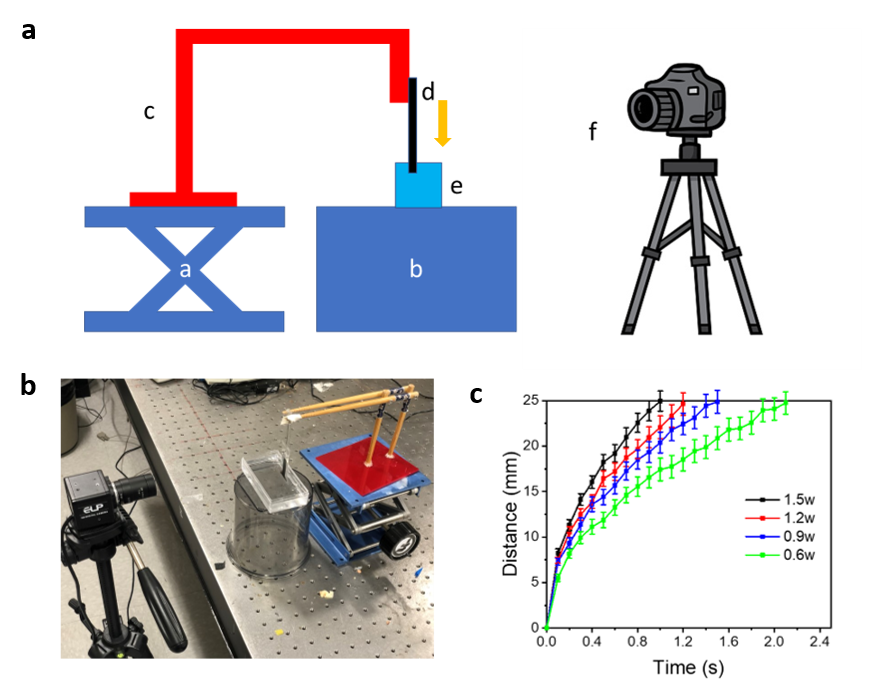


**Figure S6:** a) Experimental setup measuring wettability of the SWBM: a) z translation stage, b) mount, c) crane structure, d) SWBM, e) water reservoir, f) camera. The gold arrow represents the moving direction of the SWBM. b) Picture of the experimental setup. c) Water wet front of SWBM using different power: 0.6W, 0.9W, 1.2W and 1.5W, d) Snapshots of water wicking dynamics on the surface of different SWBM. White dashed line shows the position of the water-wet front. Each error bar represents the standard deviation of mean calculated from at least 15 data points.


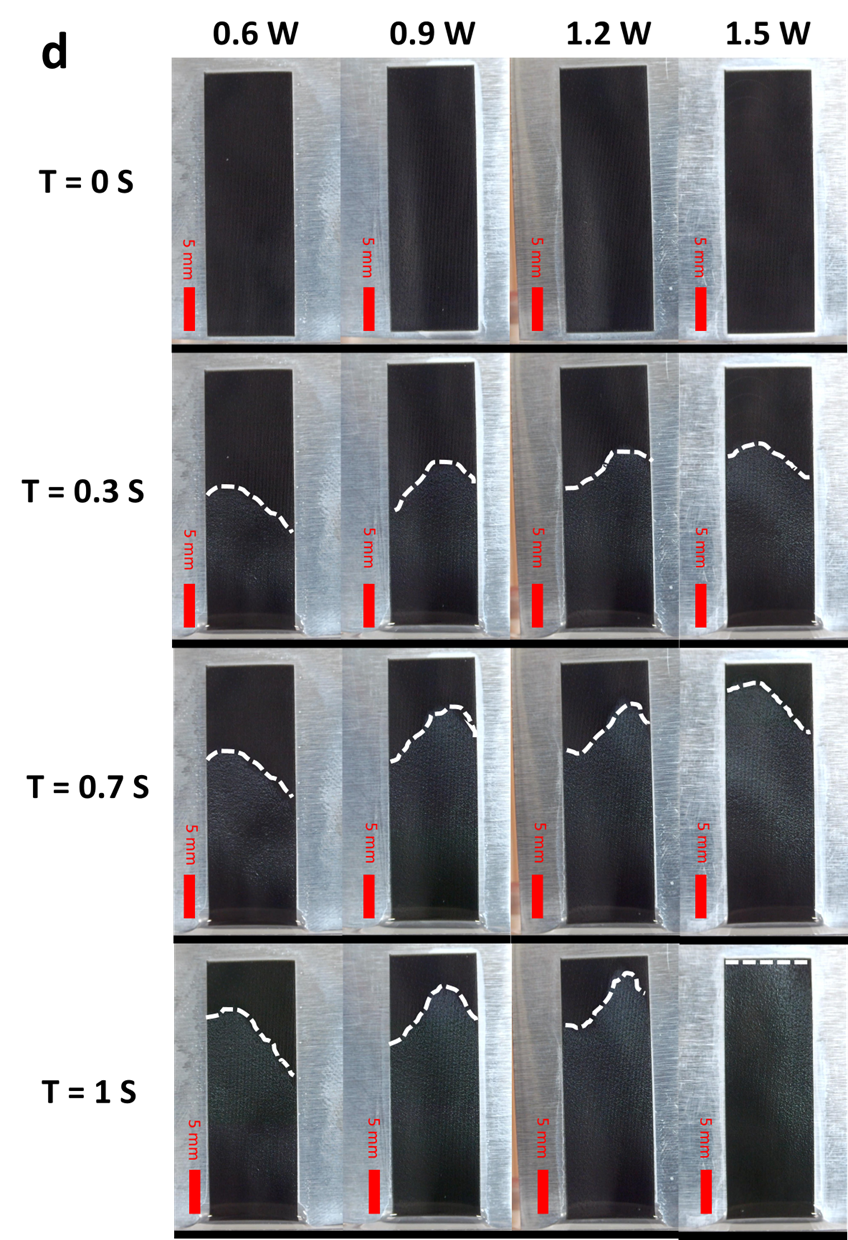


**Estimation of the amount of water supply**

The mass of water wicking the surface is calculated with the following equation:

$$\begin{aligned} M_{s}=n\times\rho\times L\times A_{s} \times f\#\left( 1 \right) \end{aligned}$$

where n is the number of capillary, $\rho$ is the density of water, $L$ is the wet front position on the SWBM, $A_{s}$ is the cross-section area of the SWBM and $f$ is the factor describes how well water fills the capillary. In this estimation, $f=1$. The wet front verse time is shown in **Fig. S5c,** and the cross-section can be calculated with the surface profile (**Fig. S4a**).


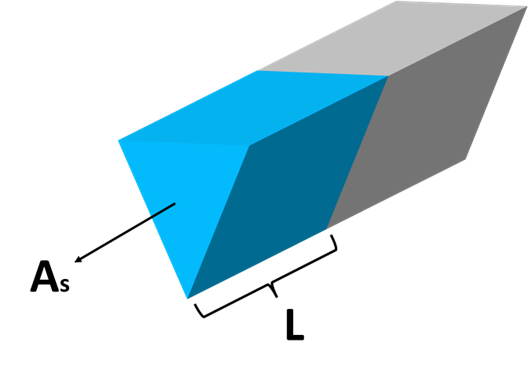


**Figure S7**: geometry model to calculate the mass of water inside the capillary.

**Supplementary Note 5**

**Broadband optical absorption of SWBM**

The broadband optical absorption of SWBM is a combined effect of surface plasmon resonance (SPR) absorption, hybridization between different SPR modes, and multiple reflections from walls of microgrooves ^2-4^. After laser processing, SWBM surface are covered with micro-and nanoparticles of sizes ranging from few nanometers to several micrometers. These wide range of micro-and nanoparticles are key components for the broadband optical absorption for SWBM. Laser power can control the density of different sizes of nanoparticles (NPs), thus determining the appearance of the produced samples (**Figure. S8**). For example, smaller size of NPs is generally produced at lower laser power and vice versa.

**Figure S8** shows the SEM images of the micro/nanostructures of the SWBM fabricated by different laser power. The density distribution of micro/nanostructures is different for SWBM samples produced at different laser power. For SWBM-0.6 and SWBM-0.9, smaller sized nanopartilces/nanostructures are dominant because nanostructures are more favorable to be formed under low ablation fluence, which has been referred to as “gentle ablation” ^5^. For SWBM-1.5, nanostructures become sparse, and microstructures are dominant because nanoparticles are more likely to be melted under high temperature caused by high laser processing power. SWBM-1.2 turns out to have the highest broadband optical absorption because it has the optimum combination of microstructures and nanostructures over a range of sizes.


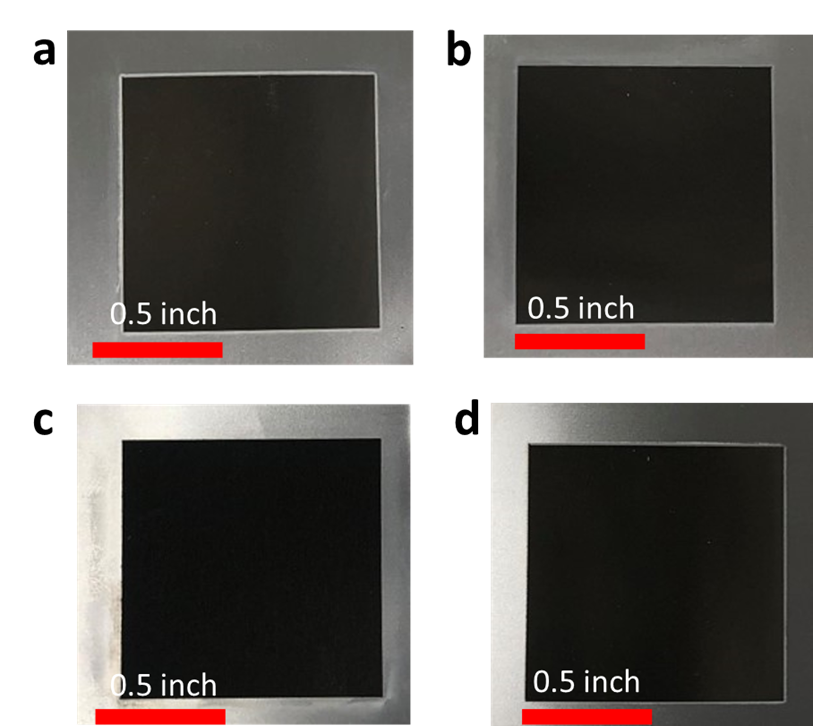


**Figure S8**: Photographs of (a) SWBM-0.6, (b) SWBM-0.9, (c) SWBM-1.2, and (d) SWBM-1.5 samples. Scale bar is 0.5 inch for all images.

**
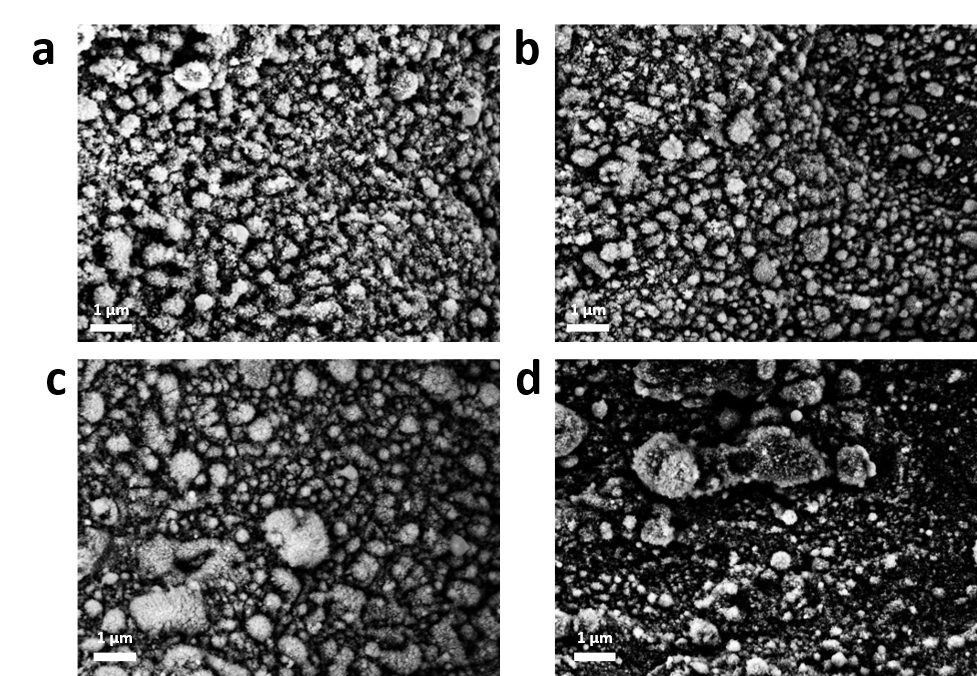
**

**Figure S9**: SEM images of (a) SWBM-0.6, (b) SWBM-0.9, (c) SWBM-1.2, and (d) SWBM-1.5 samples. Scale bar is 1 micron for all images.

**Experiment setup measuring evaporation rate of SWBM**

**
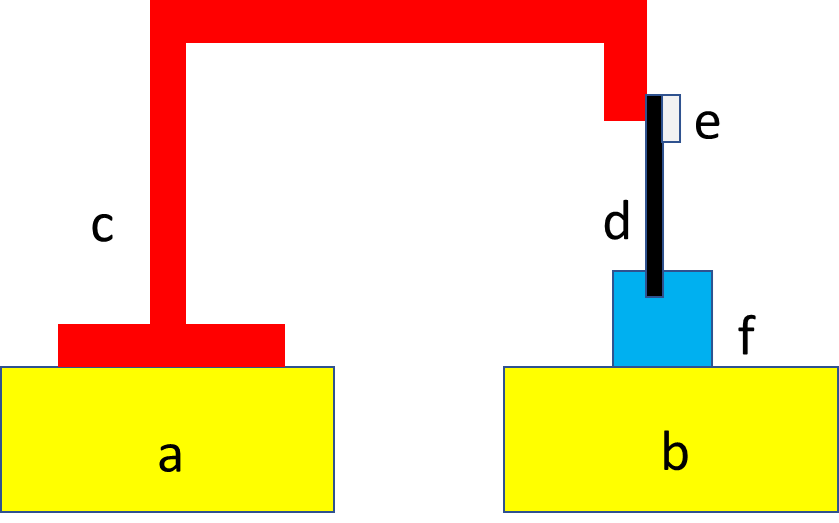
**

**Figure S10**: Experiment setup schematic diagram measuring the solar thermal evaporation rate of different SWBM: a) table, b) balance, c) jib crane structure, d) SWBM, e) salt harvested, f) water reservoir.

**Supplementary Note 6**

**Overall design of desalination surface architecture**

For the surface design of the SWBM desalination system, some surrounding passive areas are left deliberately. The main purpose of this architecture is for convenience of the experiment as we are able to observe the laser treated area and salt creeping process from the front side at the same time. If the surrounding passive area is cut, we need to observe both the front side and the back side of the SWBM as salt crystal expands on the backside passive area now. **Figure. S11** shows the snapshot of the front side and back side of the SWBM without surrounding passive area under one sun illumination treating ocean water for 2 hours and 8 hours. The front surface exhibits self-cleaning property while salt accumulates on the back passive area. Therefore, the surrounding passive area is left for convenience of the experiment and has no effect to the self-cleaning property of the system.

**
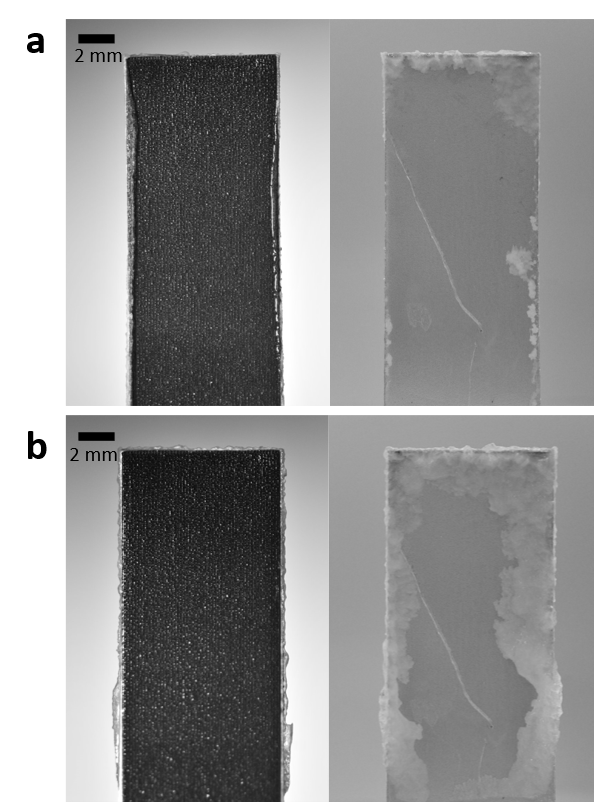
**

**Figure S11**: Snap shots of the front and back side of SWBM when treating ocean water under one sun illumination for (a) 2 hour and (b) 8 hours.

**Supplementary Note 7**

**Water meniscus profile in the micro capillary**

The water meniscus profile in the micro capillary is measured using the UV laser microscope. **Figure S12** shows the experiment setup. The SWBM is mounted beneath the microscope so that the cross section of the top part of the SWBM can be directly examined by the microscope. **Figure S13** displays the water meniscus profile of SWBM-1.2 and SWBM-1.5 when water is supplied. The meniscus profile for SWBM becomes more flatten when the laser processing power increases. For SWBM made by higher laser power, water wicks the micro grooves easier (**Figure S6c**), resulting in more volume of water filled in the micro grooves. Therefore, the air-water interface in the micro grooves decreases with the laser processing power.


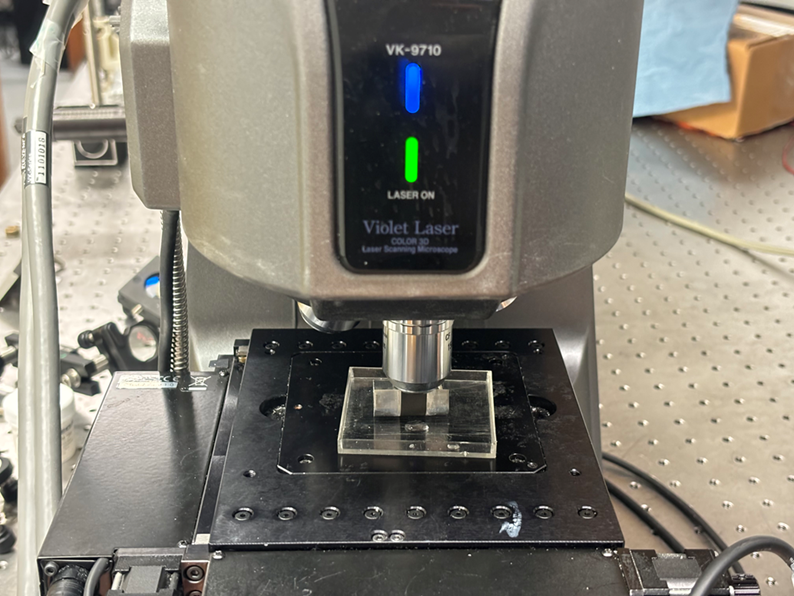


**Figure S12**: Experiment setup to measure the water meniscus profile using microscope.


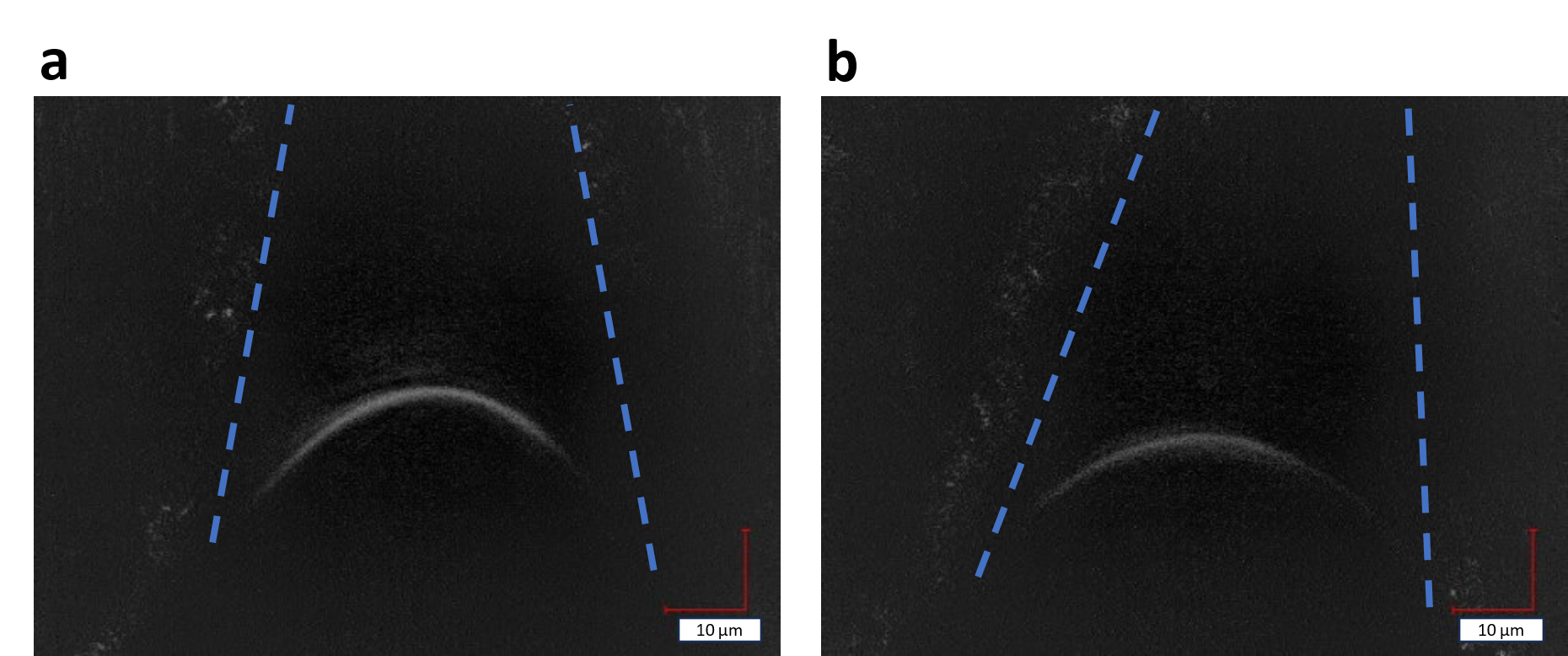


**Figure S13**: Image showing the water meniscus profile (a) SWBM-1.2, and (b) SWBM-1.5 samples. Scale bar is 10 microns for all images. The blue dot line marked the surface profile of the micro grooves.

**Supplementary Note 8**

**Spatial variation in water transport rate**

The spatial variation in water transport can be evidenced by the water boundary shown in the **Fig. 2c**. The water profile is about parabola which means that the water wicking speed and resulting water transport rate is higher at the center and lower at the edge. This spatial variation originates from the characteristic of the laminar flow. The water flow in each micro capillary is not independent but interconnected with each other. Therefore, the water flow on the surface could be treated as a laminar flow. The Reynolds number (*RE*) for this flow could be estimated as:

$$\begin{aligned} RE=\frac{\rho uL}{\mu}=0.249 \#\left( 2 \right) \end{aligned}$$

where $\rho$ is the density of the fluid, $u$ is the axial velocity of the fluid, $L$ is the characterizing length of the flow, and $\mu$ is the dynamic viscosity of the fluid. In fluid dynamics, *RE* < 2500 is characterized as laminar flow. Therefore, it is accurate to treat the water flow on SWBM as laminar flow.

The velocity profile for Laminar flow can be mathematically interpreted as (**Figure S14**):

$$\begin{aligned} V\left( r \right)=\frac{1}{4\eta}\frac{dP}{dx} R^{2}\left( 1-\frac{r^{2}}{R^{2}} \right)+V\left( R \right) \#\left( 3 \right) \end{aligned}$$

where $\frac{dP}{dx}$ is the pressure gradient along axial direction, $\eta$ is the static viscosity of the fluid. As a result, water travels faster at the center and slower at the edge, resulting the spatial variation of the water transport rate.


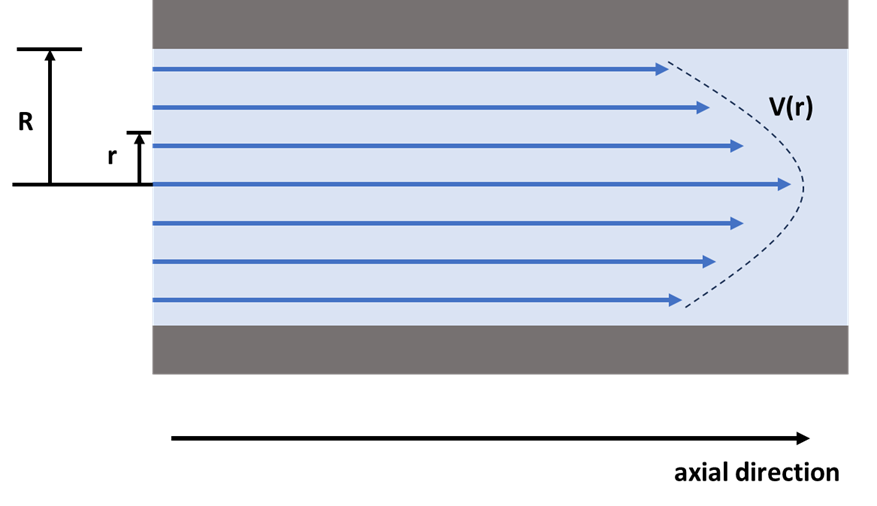


**Figure S14:** Schematic of velocity profile for a typical laminar flow.

**Supplementary Note 9**

**Solar thermal interfacial crystallizer energy efficiency calculation**

**i) Theoretical calculation of energy efficiency**

The solar evaporation efficiency is calculated via the following formula which can be found in most literature^6,7^:

$$\begin{aligned} \eta=\frac{P_{l}+P_{s}}{P_{in}} \#\left( 4 \right) \end{aligned}$$

$$\begin{aligned} P_{l}=\dot{m}\times L\left( T_{i} \right)\#\left( 5 \right) \end{aligned}$$

$$\begin{aligned} P_{s}=\dot{m}\times c\times\left( T_{i}-T_{w} \right) \#\left( 6 \right) \end{aligned}$$

where $P_{l}$ is the heating power due to latent heat of water evaporation, $P_{s}$ is the heating power due to sensitive heat of water evaporation process, $P_{in}$ is the incident simulated solar power.

Under one sun illumination, P_in_ = 1 kW m^-2^. Based on experiment results, the average evaporation rate of the SWBM when treating with real ocean water is 1.76 kg m^-2^ h^-1^. In dark time, the rate of evaporation for SWBM is 0.67 kg m^-2^ h^-^1, The net water evaporation rate will then be 1.09 kg m^-2^ h^-1^, which is the difference between solar time and dark time. When calculating the enthalpy of evaporation of seawater, we can do an approximation by treating seawater as an idea solution. Then, the enthalpy of evaporation of seawater is equal to that of pure water. The heat capacity slightly decreases when salinity increases because salt ions in seawater can disrupt the hydrogen bonds between water molecules absorbing heat. From **Figure S15**, the temperature of SWBM can be approximated as 32 ^o^C and the temperature of water is 22.5 ^o^C. At 32 ^o^C, the heat capacity of seawater is 4.003 KJ Kg^-1^ K^-1^ and latent heat is 2406 KJ Kg^-1^ ^8^. After calculation, the apparent efficiency under one sun illumination is 119.93% and net efficiency is 74.27%.

**ii) Temperature measurement of Superwicking Black Metal and water under one sun**

The experiment setup is the same as the experiment setup for evaporation measurement (**see Fig. S11**) except additional thermocouples are mounted to measure the temperature of the SWBM, water and ambient air. Under one sun illumination, the temperature of SWBM increases rapidly in the first ten minutes. It reaches 31 ^o^C from 21 ^o^C. Then, it slowly increases as time goes on, reaching 32 C^o^ after 8 hours. The water reservoir temperature just increases from 22 ^o^C to 23 ^o^C as most of the energy received is used for interfacial evaporation and the heat loss to water reservoir is small.


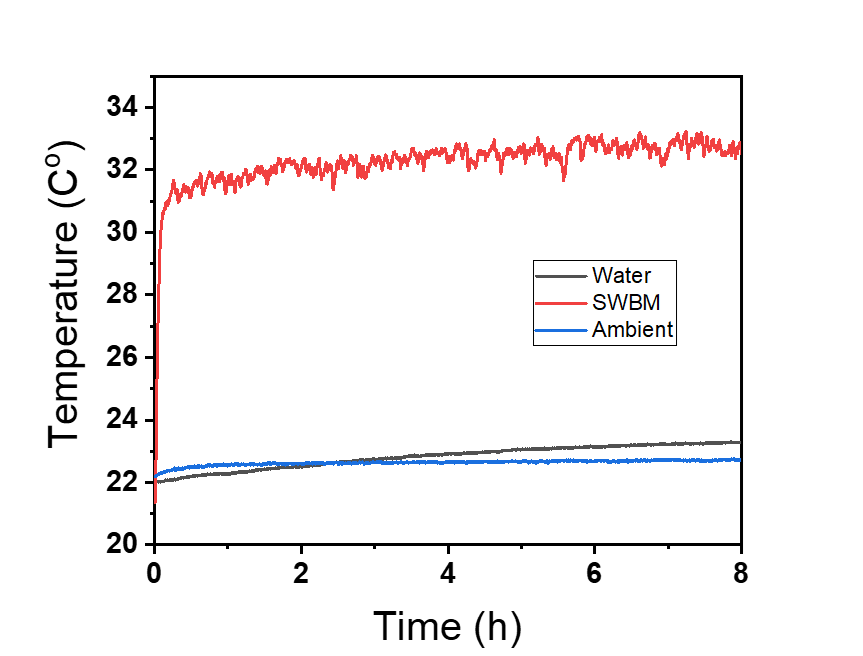


**Figure S15**: Temperature of the SWBM, ocean water reservoir and ambient under one sun illumination for 8 hours.

**Supplementary Note 10**

**Experimental design for operando mineral mining and reservoir water salinity measurements**

**i) Experiment layout**

A two-balance experiment setup is designed to record the amount of evaporation and harvested salt simultaneously. **Figure S16** shows the diagram of the experiment setup. Two balances are mounted firmly on the table. On the left balance, there is a jib crane structure. The SWBM hangs on the hook of the jib crane structure. The bottom of the SWBM contacts the water reservoir, which is on the second balance. Note that the PPM meter is not shown in the diagram for simplification because the PPM meter is incorporated with the water reservoir.

**
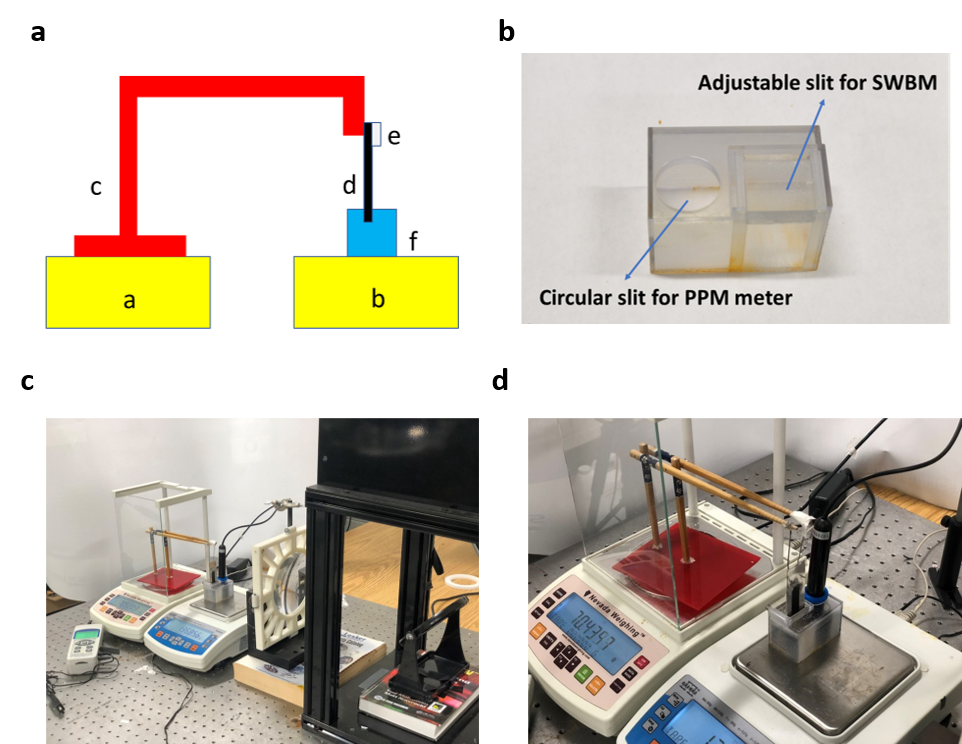
**

**Figure S16**: a) Two balance experiment setup schematic diagram: a) balance1, b) balance 2, c) jib crane structure, d) SWBM, e) salt harvested, f) water reservoir. b) 3D printed reservoir with a circular aperture to incorporate PPM meter. c) Image of two balance experiment setup. The lens could be integrated for high solar concentration conditions. d) Zoom view of two balances.

**ii) Force analysis of the system**

**Figure S17** shows the force diagrams of the crane structure, the SWBM, and water reservoir. The force equations for these three objects are listed below:

$$\begin{aligned} F_{b1}-F_{gc}-F_{cs}=0 \#\left( 7 \right) \end{aligned}$$

where $F_{b1}$ is the normal force between balance 1 and the crane structure, $F_{gc}$ is the gravity force of the crane structure and $F_{cs}$ is the normal force between the crane structure and the SWBM.

$$\begin{aligned} F_{cs}+F_{b}-F_{ss}-F_{gs}-F_{sw}=0 \#\left( 8 \right) \end{aligned}$$

where $F_{b}$ is the buoyant force, $F_{ss}$ is the friction force between the SWBM and the salt sticking on its surface. The magnitude is equivalent to the gravity force of the salt. $F_{gs}$ is the gravity force of SWBM and $F_{sw}$ is the hydrophilic force between the SWBM and water.

$$\begin{aligned} F_{b2}+F_{sw}-F_{gw}-F_{b}=0 \#\left( 9 \right) \end{aligned}$$

where $F_{b2}$ is the normal force between balance 2 and water and $F_{gw}$ is the gravity force of water.

During the experiment time, water evaporates, and salt crystallizes on the surface of SWBM. The changing of gravity force of water and salt can be represented by the following two equations:

$$\begin{aligned} \Delta F_{gw}=\Delta m_{w}g +\Delta m_{w}\rho g\#\left( 10 \right) \end{aligned}$$

$$\begin{aligned} \Delta F_{ss}=-\Delta m_{w}\rho g \#\left( 11 \right) \end{aligned}$$

where $\Delta m_{w}$ is the mass change of water due to evaporation, $\rho$ is PPM of the water and $g$ is gravity constant.

The water level drops because of the evaporation loss. Since this process is gradually, the system can still be regarded in a stable condition: the SWBM hangs firmly on the hook of jib crane structure and water surface is calm. Therefore, the hydrophilic force between SWBM and water doesn’t change with time.

$$\begin{aligned} \Delta F_{sw}=0 \#\left( 12 \right) \end{aligned}$$

Buoyant force slightly decreases because the water level drops due to the evaporation loss. The decrease of buoyant force can be calculated as:

$$\begin{aligned} \Delta F_{b}=\Delta m_{w}\frac{A_{S}}{A_{W}}g \#\left( 13 \right) \end{aligned}$$

where $\Delta m_{w}$ is the mass change of water due to evaporation, $A_{S}$ is the cross-section area of SWBM, $A_{W}$ is the area of water reservoir.

Now, combining equations above, we can get:

$$\begin{aligned} \Delta F_{b1}=\Delta F_{ss}-\Delta F_{b}=-\Delta m_{w}\rho g-\Delta m_{w}\frac{A_{S}}{A_{W}}g \#\left( 14 \right) \end{aligned}$$

$$\begin{aligned} \Delta F_{b2}=\Delta F_{gw}+\Delta F_{b}=\Delta m_{w}g+\Delta m_{w}\frac{A_{S}}{A_{W}}g \#\left( 15 \right) \end{aligned}$$

Using $A_{S}=0.02 cm^{2}$, $A_{w}=24 cm^{2}$ and $\rho=0.035$, we get $\frac{A_{S}}{A_{W}}: \rho=1:42$, Thus, the change of buoyant force is negligible compared with the change of gravity force of water and salt. We can assume:

$$\begin{aligned} \Delta F_{b}=0 \#\left( 16 \right) \end{aligned}$$

Finally, we can get:

$$\begin{aligned} \Delta F_{b1}=\Delta F_{ss}=-\Delta m_{w}\rho g \#\left( 17 \right) \end{aligned}$$

$$\begin{aligned} \Delta F_{b2}=-\Delta F_{gw}=\Delta m_{w}g +\Delta m_{w}\rho g\#\left( 18 \right) \end{aligned}$$

The result shows that the change of mass reading on balance 1 represents the accumulation of salt on the SWBM surface and the change of mass reading on balance 2 represents the loss of water and mineral in the reservoir due to evaporation.


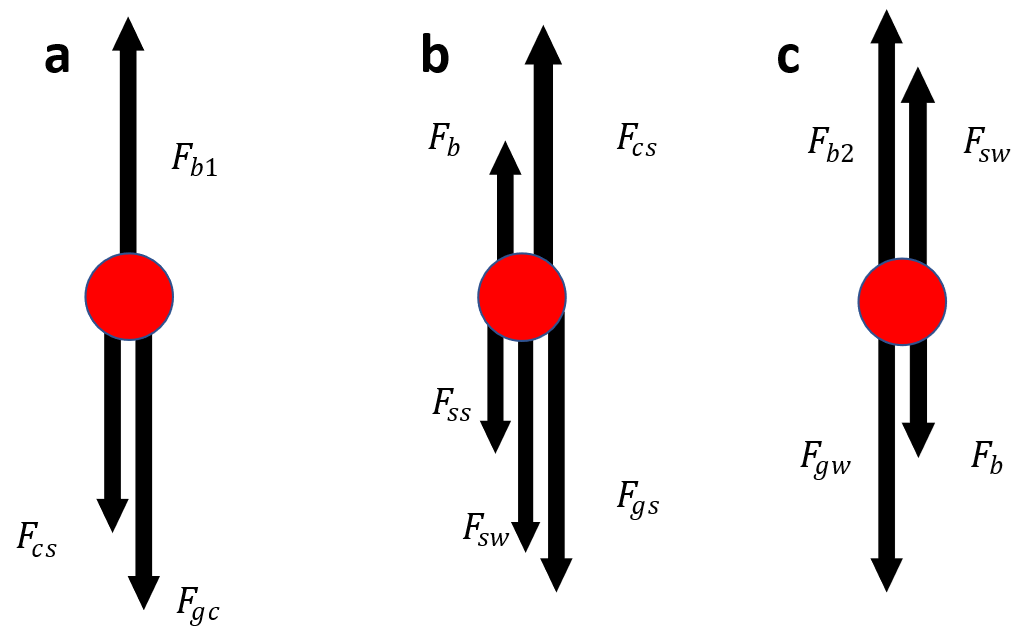


**Figure S17**: Force diagram of a) the crane structure, b) the SWBM, c) water reservoir

**Supplementary Note 11**

**Durability analysis of Superwicking Black Metal**

In summary, aging is the primary factor affecting the long-term performance of the SWBM. **Figure. 4d** in manuscript already shows good long-term performance of SWBM in the aspect of evaporation rate and salt harvesting rate. Here are several more studies for verification. The study compares the pristine SWBM and 7-day usage SWBM for three essential properties that determine SWBM's desalination performance: the solar spectrum of SWBM, its wicking property, and the maximum amount of water it can absorb.

**i) Solar spectrum comparison**

The solar spectrum determines the amount of energy SWBM can receive for STIC, while the wicking property and the maximum absorbable water determine the quality of the self-cleaning mechanism. **Figure. S18a** shows the solar absorption spectrum comparison. The absorption spectrum decreases about 2% after 7 days usage. This quantity of decrease is small, and SWBM still preserves high absorption spectrum overall.

**ii) Wicking dynamics comparison**

**Figure. S18b** presents a comparison of the wicking property of SWBM, indicating that the propagation of waterfronts is slightly affected by aging: about extra 0.1 second is needed to finish propagating 25 mm.

**iii) Maximum capacity of water absorption**

To compare the maximum absorbable water in SWBM, we introduced an experiment setup shown in **Fig. S18d**. We vertically mount the SWBM on a precise micrometer-controlled translational stage. First, we gradually lower the SWBM and let it touch the water reservoir on a weighing balance. After SWBM contacts the water surface, we wait for about 30 seconds to let SWBM absorb the maximum amount of water. Finally, we pull the SWBM out of contact with water. **Fig S18c** shows mass reading for the whole experiment process. From part A to part B, it is the time when we try to lower the SWBM to touch the water surface, but they are not touching yet. The mass decrease is totally due to natural evaporation loss. Part B to Part C represents the moment when SWBM contacts the water surface. This huge mass decrease is due to two factors: capillary force that drags water in the micro capillaries and the hydrophilic force between SWBM and water. Part C to Part D is when we wait for the system to be stable. The mass change here is also due to evaporation loss. Part D to Part E is the moment when we pull the SWBM out of the water surface. The hydrophilic force between the SWBM and water increases a little while we pull out the SWBM because water adheres to the surface strongly. Part E to Part F is when the SWBM is separated from water surface. The mass change is caused by three factors: vanish of hydrophilic force between SWBM and water, vanish of capillary force and decrease mass for water absorbed by SWBM. Part F to Part G is the final moment of the experiment. Therefore, the mass difference between AB and FG shows the amount of water absorbed by the SWBM. From **Fig. S18c**, we found that the maximum absorbable water of the SWBM nearly doesn’t change due to aging.

In conclusion, the long-term desalination performance of SWBM is guaranteed as the solar spectrum, wicking dynamics and maximum absorbable water decays pretty slow with time.


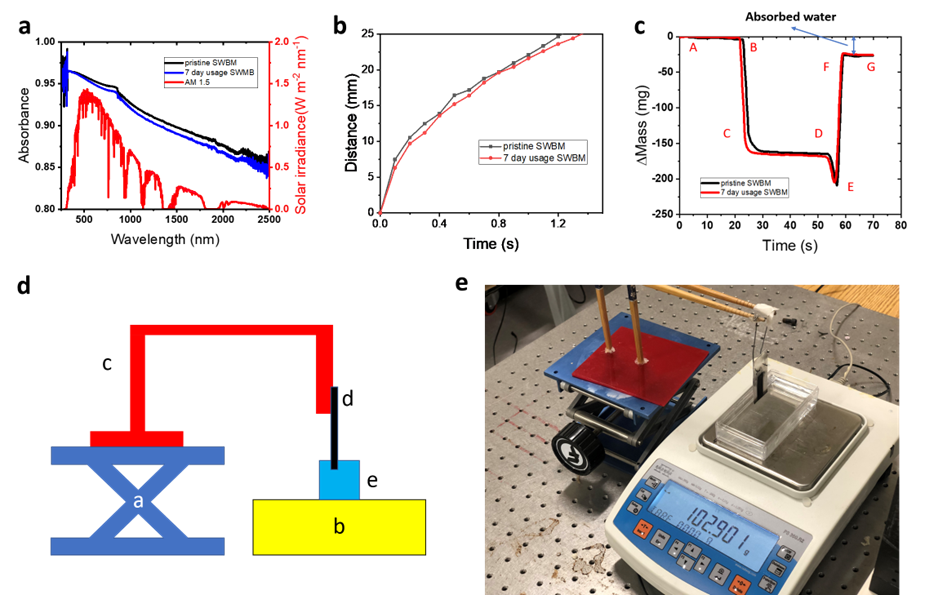


**Figure S18**: a) Solar absorption spectrum comparison between pristine SWBM and 7 day usage SWBM, b) Wicking dynamic comparison between pristine SWBM and 7 day usage SWBM, c) Mass reading from the balance for the experiment measuring maximum amount of water absorbed by SWBM. A-B: SWBM lowering down before touching water surface, B-C: the moment that SWBM AL touches water surface, C-D: wait for system to stabilize, D-E: try to pull SWAL AL out of the water surface, E-F: the moment that SWBM AL and water surface separates, F-G: end of experiment. d) Setup measuring maximum amount of water SWBM can absorb, a) z translation stage, b) balance, c) jib crane structure, d) SWSBM, e) water reservoir.

**Supplementary Note 12**

**Elemental analysis of mined mineral**

The seasalt harvested by ABF-STIC is analyzed by EDAX spectroscopy. **Figure. S19** displays the distribution of ions for each element. The results indicate that the predominant element in the sample is sodium, which is consistent with the fact that sea salt is primarily composed of sodium chloride. Additionally, there are detectable clusters of magnesium, potassium, and calcium ions. This observation suggests the presence of salt crystals based on magnesium, potassium, and calcium mixed in with sodium-based salt. Furthermore, bright nano spots in EDX image show the presence of valuable rare earth elements, such as uranium, cesium, bromine, and gold. Unlike sodium and other dominant sea salt elements, these rare earth elements are dispersed in nanoscale regions in the harvested seasalt. We estimated size of these nanoscale regions using Image J software. The size of these regions is calculated based on the dimension of the illuminated pixels and the scaling factor of the image. **Figure. S20c** illustrates the histogram of these nanoscale regions with their sizes primarily falling in a range of 200 to 500 nanometers.”


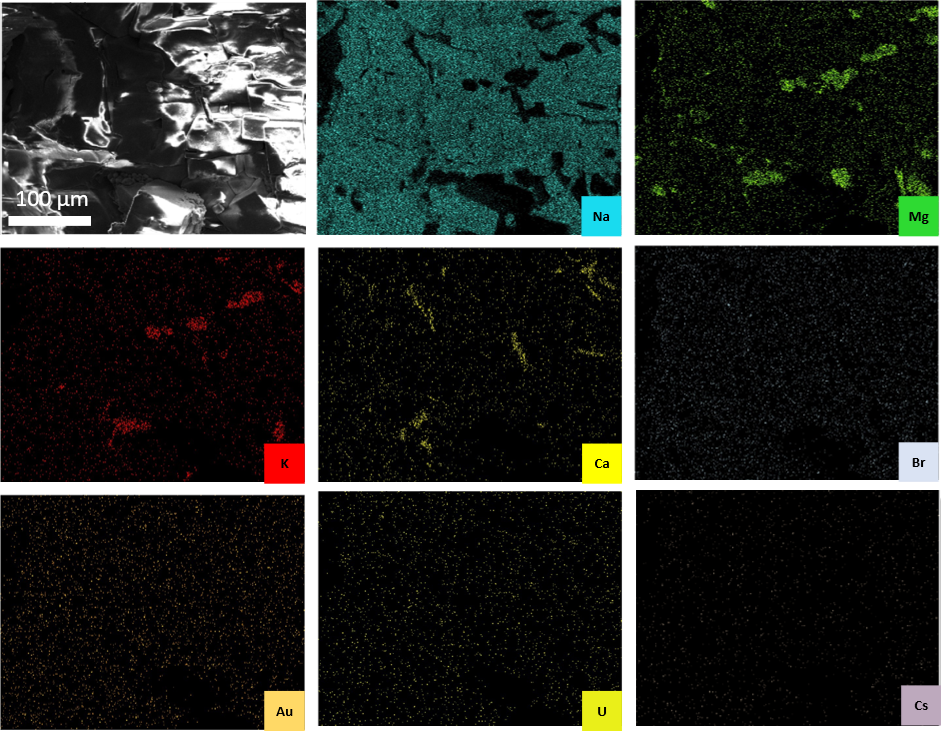


**Figure S19**: EDX result of sea salt with ion distribution of sodium, magnesium, potassium, calcium, bromine, gold, uranium, and cesium.

**
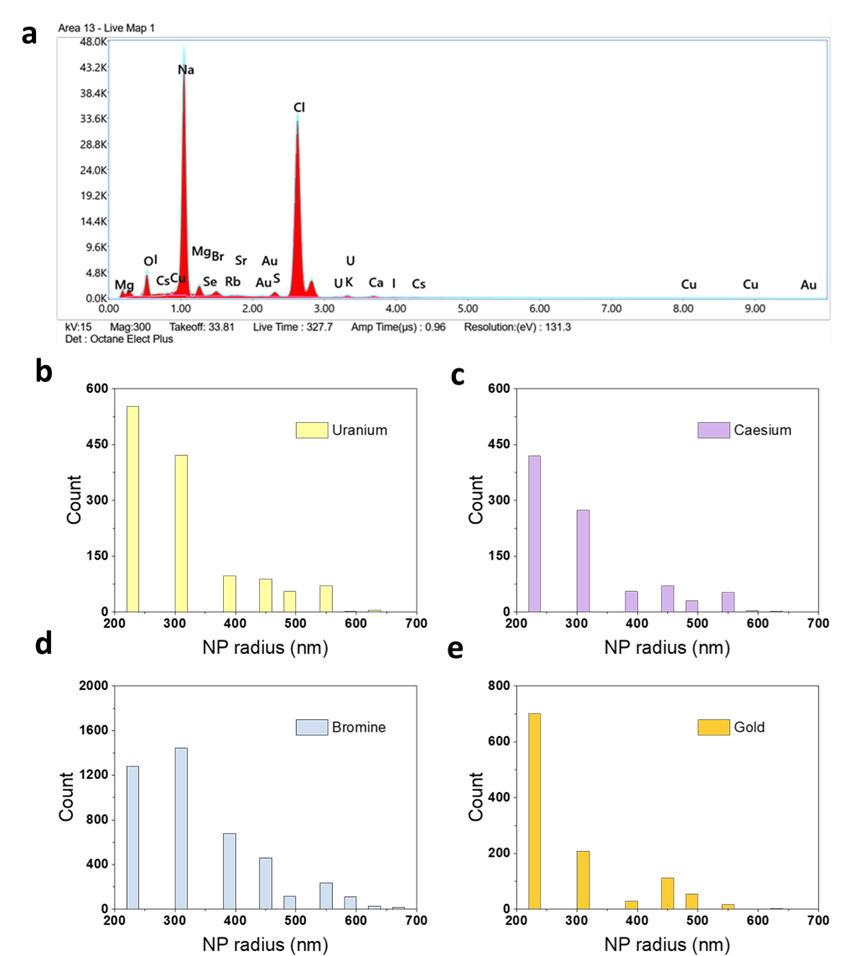
**

**Figure S20**: a) Spectrum intensity of different element measured by EDAX spectroscopy, b-d), Histogram of radius of nanoparticles of Uranium, Cesium, Bromine and Gold based on image processing estimation.

**Lithium ion mining from ocean water**

The salt harvested by ABF-STIC without further separation/purification may find limited use. Here, we demonstrate the lithium extraction using ABF-STIC. The selective lithium-ion extraction is made possible through functionalizing ABF-STIC microcapillary surfaces by depositing metatitanic acid (HTO) nanoparticles (**Figure S21a**). The lithium ions from oceanwater are selectively trapped by HTO nanoparticles in the active area during the desalination process. Later, Li can be harvested using an acid treatment (**Figure S21b**). The reactions responsible for lithium mining are as below during desalination.

H_2_TiO_3_+ 2Li^+^(seawater)→ Li_2_TiO_3_+2H^+^

Li_2_TiO_3_+ 2H^+^(acid)→ H_2_TiO_3_+2Li^+^


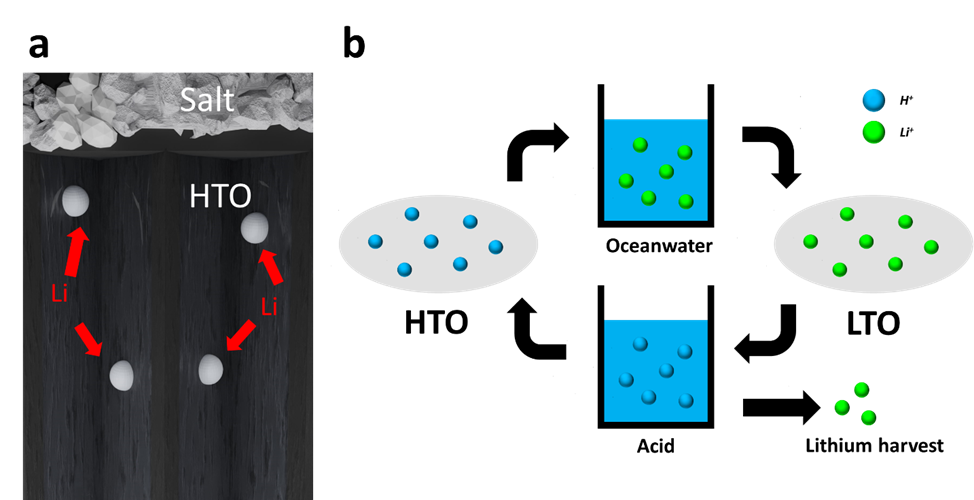


**Figure S21**: (a) Schematics showing the working principle of the lithium-ion sieve technology. (b) Schematics showing the working principle of the lithium-ion sieve technology.

To test the lithium-ion harvesting performance of ABF-STIC, we run the device continuously over 24 hours under one 1-sun solar irradiation treating ocean water. The evidence of lithium ions accumulated on the active regions of the ABF-STIC was shown using laser induced breakdown spectroscopy (LIBS). **Figure S22b** shows the microscopic image of the active region of SWBM. The white particles are the HTO coated in the micro capillaries. The red dots represent the location for LIBS analysis. **Figure S22c** is the corresponding LIBS spectrum. The presence of atomic lines at 610 nm and 670 nm are the evidence for presence of lithium^9^. Other atomic lines around 335, 455 and 498 nm wavelengths are from Ti and O from HTO ^10-13^. These demonstrations shows that other minerals can also be similarly mined from seawater during desalination. For example, TiO_2_ molecules can be coated at the active regions of ABF-STIC for uranium harvesting ^14^.

Lithium extraction from the SWBM surface is relatively straightforward. The process involves immersing the used SWBM in a dilute hydrochloric acid (HCl) solution, which effectively elutes the adsorbed lithium ions from the surface. Unlike solvent extraction or ion-exchange methods, this simple acid leaching approach minimizes operational complexity and avoids secondary chemical waste. The eluate consists primarily of lithium chloride in dilute acid, which can be neutralized and managed with standard procedures, posing minimal environmental risks. This simplicity makes the lithium extraction step both technically and environmentally feasible, though optimizing recovery yield and integrating closed-loop acid recycling will be important for large-scale applications.


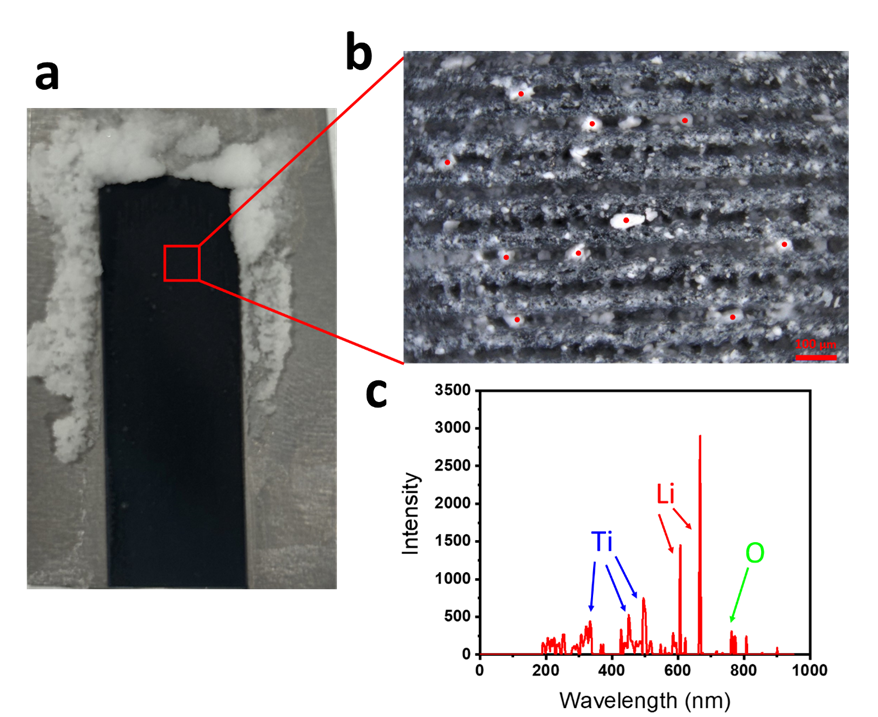


**Figure S22**: (a) self-cleaning SWBM after interfacial crystallization. (b) Image of the microcapillary of SWBM coated with HTO. Red spot represents the location for LIBS analysis. (c) LIBS spectrum of the HTO showing the Lithium harvesting.

**Supplementary Note 13**

**Mineral mining and self-cleaning dynamics**

**Observation of salt growth using microscope**

Due to the vertical orientation of the microscope objectives, it is not feasible to directly observe the salt growth patterns when the SWBM is mounted vertically. Therefore, an altered configuration, as depicted in **Figure. S23a**, is employed. In this setup, the SWBM is placed horizontally on the microscope's platform. Adjacent to the microscope, a solar simulator is installed at an angle, with its power adjusted to produce irradiance equivalent to one sun. Seawater is consistently introduced from the bottom of the SWBM using a syringe. This arrangement replicates the experimental conditions as closely as possible, allowing us to observe the salt growth phenomenon, which can subsequently be applied to experimental scenarios.

**Outward and inward salt expansion for different SWBM**

**Figure S23 b** top illustrates a time lapse of the magnified view of the salt growing process on SWBM-1.2 by focusing on a part of the saltwater boundary. Initially, salt crystallizes at the saltwater boundary just outside of the active region (marked by blue dashed line), then advances ahead forming a separate salt boundary in the passive region (marked by red dashed line). As time goes on, both the saltwater boundary (blue dashed line) and the salt boundary (red dashed line) expand outward. As a result, no salt crystal is observed in the active region. The bottom three images illustrate the time lapse for SWBM-0.6. Inward salt expansion is observed due to insufficient solution dissolving strength.

**Thin film solution wicking on salt crystals**

The thin film solution wicking on salt crystals is directly observed using light microscope. Firstly, seawater is supplied from the bottom of the SWBM and waits for natural drying. Sea salt crystals will form on the passive area due to the coffee ring effect. **Fig. S23b** shows the sea salt crystals on the passive area in dry condition. Subsequently, seawater is reintroduced, and as shown in **Figure. S23c**, the image becomes defocused, indicating that the salt crystals are covered by the thin film solution.

**Water transportation through pores of salt crystal**

Sea salt primarily comprises sodium chloride, whose crystal structure is cubic ^15^. The presence of imperfections in the arrangement of these cubic crystals results in the formation of tiny gaps between them, rendering sea salt porous. Consequently, thin film solution can permeate through these crystals and travel within their pores, as illustrated in **Figure. S23d**. In this figure, the grey cubes signify the salt crystals, while the blue arrow signifies the movement of water. As a consequence of this water movement, a thin layer of liquid forms on the surface of these crystals. Subsequently, water evaporates rapidly from the surface of these crystals, as depicted in **Figure. S23e**. Consequently, the overall rate of evaporation increases.


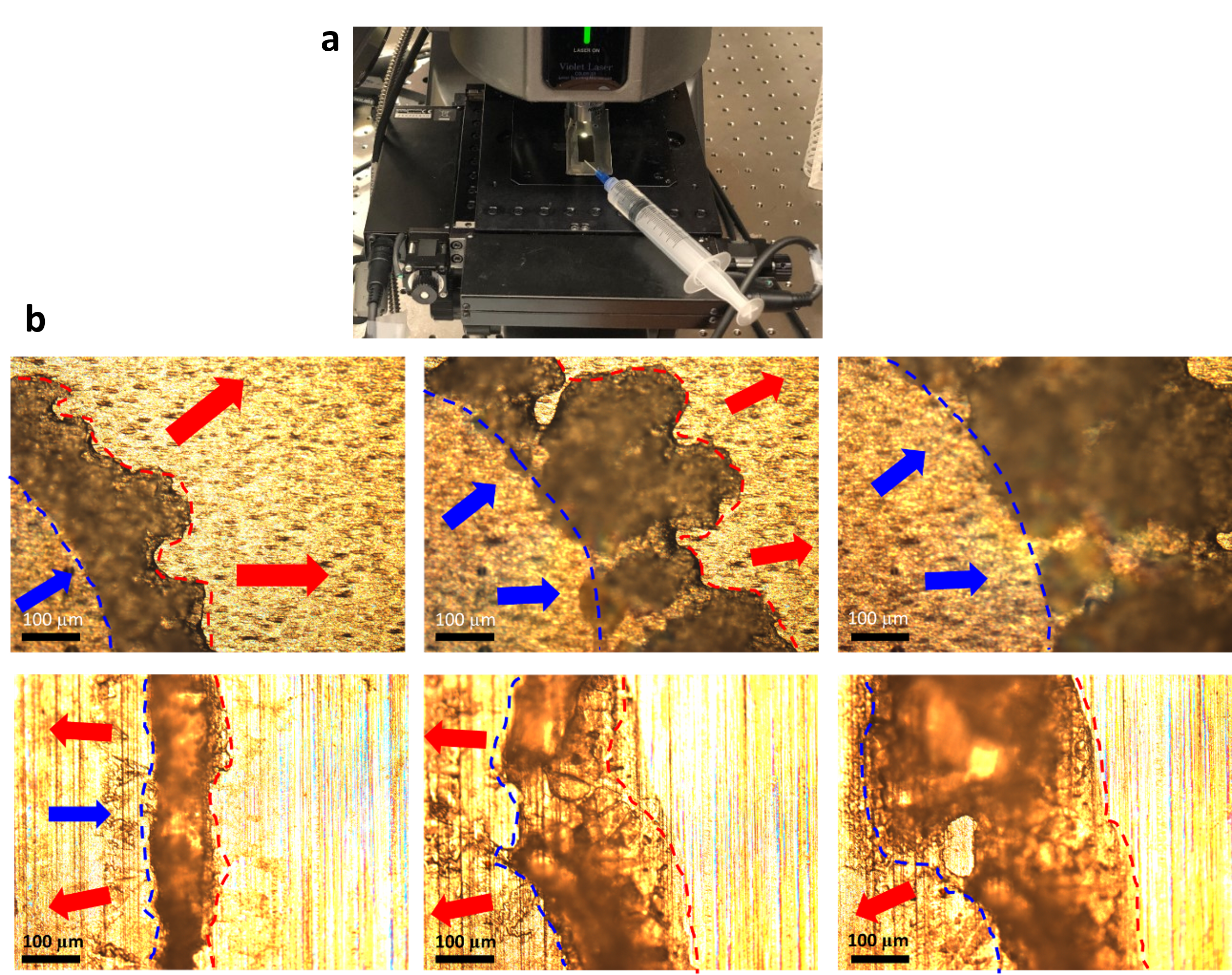

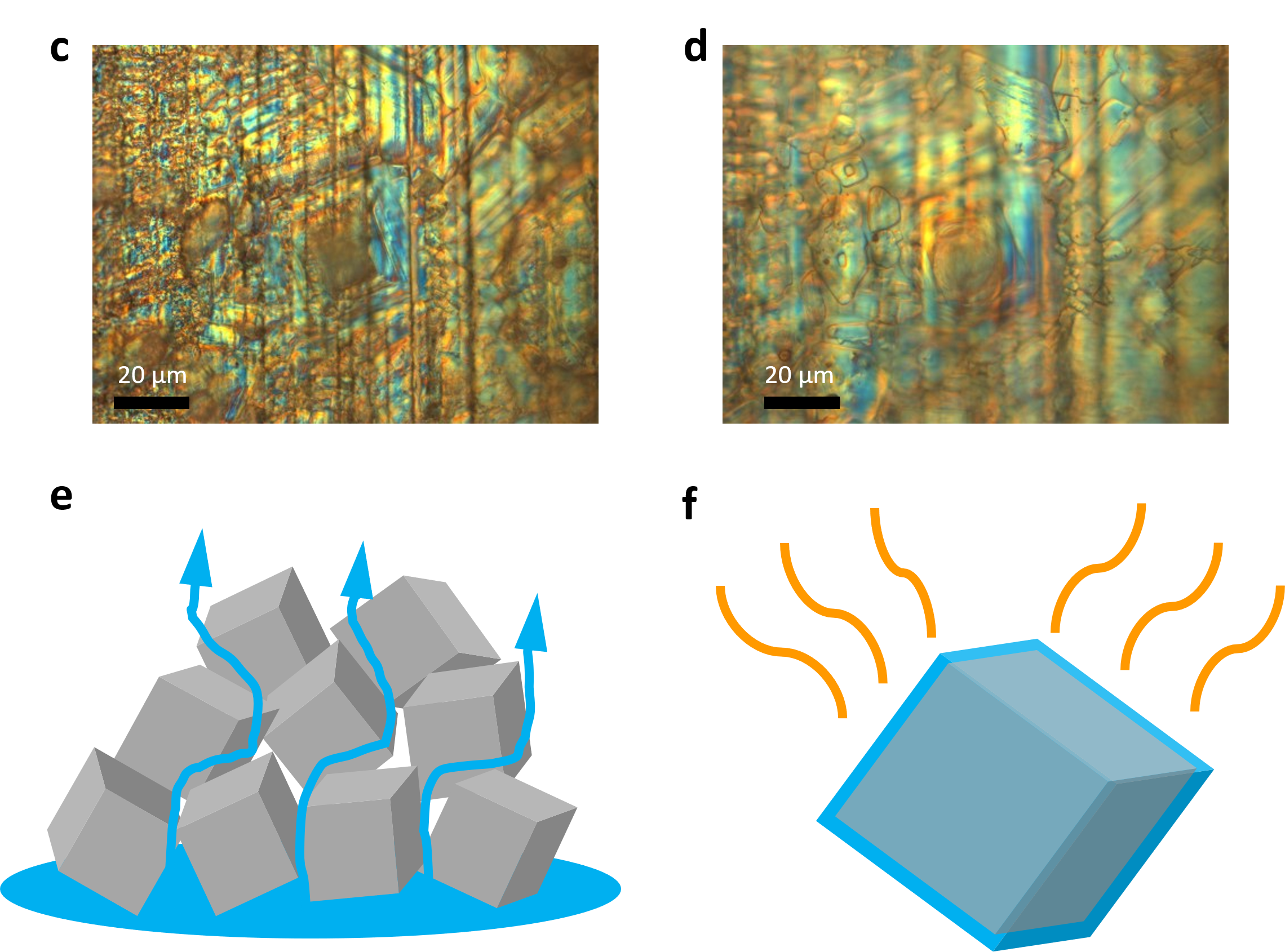


**Figure S23**: a) Experiment setup observing salt growth using light microscope, b) Time-lapse microscope view of the salt growth phenomenon over a 10-minute duration. The blue dashed lines mark the saltwater boundary, and the red dashed lines mark the salt boundary. The time interval between each image is 5 minutes. The top three images is outward salt expansion for SWBM-1.2 and bottom three image is inward salt expansion for SWBM-0.6. c) Salt crystal on the passive area in dry condition. d) Salt crystal covered by the thin film solution when water is supplied. e) Water transportation through pores of sea salt. f) Thin film forms on the salt crystal and boosts evaporation.

**Supplementary Note 14**

**Salt solution concentration distribution along the capillary**

A physics-based model is utilized to approximate the concentration distribution of the solution throughout the capillary. Only one capillary is examined assuming the uniformity of all capillaries in the SWBM. The distribution of concentration along the capillary is illustrated by a 101 x 1 matrix, where each element in the matrix represents the average concentration of an area. The first element denotes the passive region on the top, while the remaining 100 elements signify the capillary. Initially, the concentration of each element is equal to that of the water reservoir. Then, the concentration change for each unit is computed after every time step, which is 0.1 seconds. The concentration change is evaluated by considering several processes such as evaporation, dilution, ion diffusion, crystallization, and dissolution. **Figure. S24a** depicts the flow chart of how the concentration change is calculated. To simplify the calculation, several assumptions are made.

1. The rate of evaporation is the same everywhere in the capillary.
2. The evaporation loss is balanced by the upward capillary flow quickly so that the volume of water in the capillary doesn’t change.
3. The capillary shape profile is uniform along the capillary so that the volume of each unit is the same.
4. The coefficient of ion diffusion and dissolution is the same everywhere.
5. Salt participates quickly as long as concentration is larger than saturation.

Details of how each mechanism change concentration values are shown below:

Evaporation:

$$\begin{aligned} C_{i}=\left\{ \begin{aligned} \\ \frac{C_{i}\times V}{V-bkt} \left( i=1 \right) \\ \frac{C_{i}\times V}{V-kt} \left( else \right) \end{aligned} \right. \#\left( 19 \right) \end{aligned}$$

Dilution:

$$\begin{aligned} C_{i}=\left\{ \begin{aligned} \\ \frac{C_{i}\times\left( V-\left( i+b \right)kt \right)+C_{w}\times\left( i+b \right)kt}{V} \left( i=101 \right) \\ \frac{C_{i}\times\left( V-\left( i+b \right)kt \right)+C_{i+1}\times\left( i+b \right)kt}{V} \left( else \right) \end{aligned} \right. \#\left( 20 \right) \end{aligned}$$

Ion diffusion^16^:

$$\begin{aligned} C_{i}=\left\{ \begin{aligned} \frac{{[C}_{i}\times V-\frac{\left( C_{i}-C_{i-1} \right)}{L}\times k_{i}t]}{V} \left( i=1 \right) \\ \frac{{[C}_{i}\times V-\frac{\left( {2C}_{i}-C_{w}-C_{i-1} \right)}{L}\times k_{i}t]}{V} \left( i=101 \right) \\ \frac{{[C}_{i}\times V-\frac{\left( {2C}_{i}-C_{i+1}-C_{i-1} \right)}{L}\times k_{i}t]}{V} \left( else \right) \end{aligned} \right. \#\left( 21 \right) \end{aligned}$$

Crystallization:

$$\begin{aligned} if C_{i}\geq C_{s}, then\left\{ \begin{aligned} C_{i}=C_{s} \\ P_{i}=P_{i}+\left( C_{i}-C_{s} \right)\times Vt \end{aligned} \right. \#\left( 22 \right) \end{aligned}$$

Dissolution^17^:

$$\begin{aligned} if P_{i-1} \geq0, then\left\{ \begin{aligned} C_{i}=\frac{C_{i}\times V+\frac{\left( C_{s}-C_{i} \right)}{C_{s}}\times k_{d}t}{V} \\ P_{i-1}=P_{i-1}-\frac{\left( C_{s}-C_{i} \right)}{C_{s}}\times k_{d}t \end{aligned} \right. \#\left( 23 \right) \end{aligned}$$

where $C_{i}$ is the concentration in ith unit, $C_{w}$ is the concentration of water reservoir, $C_{s}$ is the saturation concentration of water reservoir, $V$ is the volume of each unit, $L$is the length of each unit, $t$is time step, $b$ is the scaling factor for passive area, $S$ is the cross section area of the capillary, $k$is the rate of evaporation, $k_{i}$ is the rate of ion diffusion in capillary, $k_{d}$is the rate of dissolving and $P_{i}$ is the volume of crystallization in ith unit.

**Figure. S24b** displays stable concentration distribution. The concentration exhibits an exponential distribution along the capillary. In stable condition, only the first unit’s concentration becomes higher than saturation, which means salt only crystallized on the passive area. The concentration close to the water reservoir has nearly the same concentration as the water reservoir. Therefore, the ion diffusion back to the water reservoir is negligible and all the ions finally crystallized on the passive area.

**
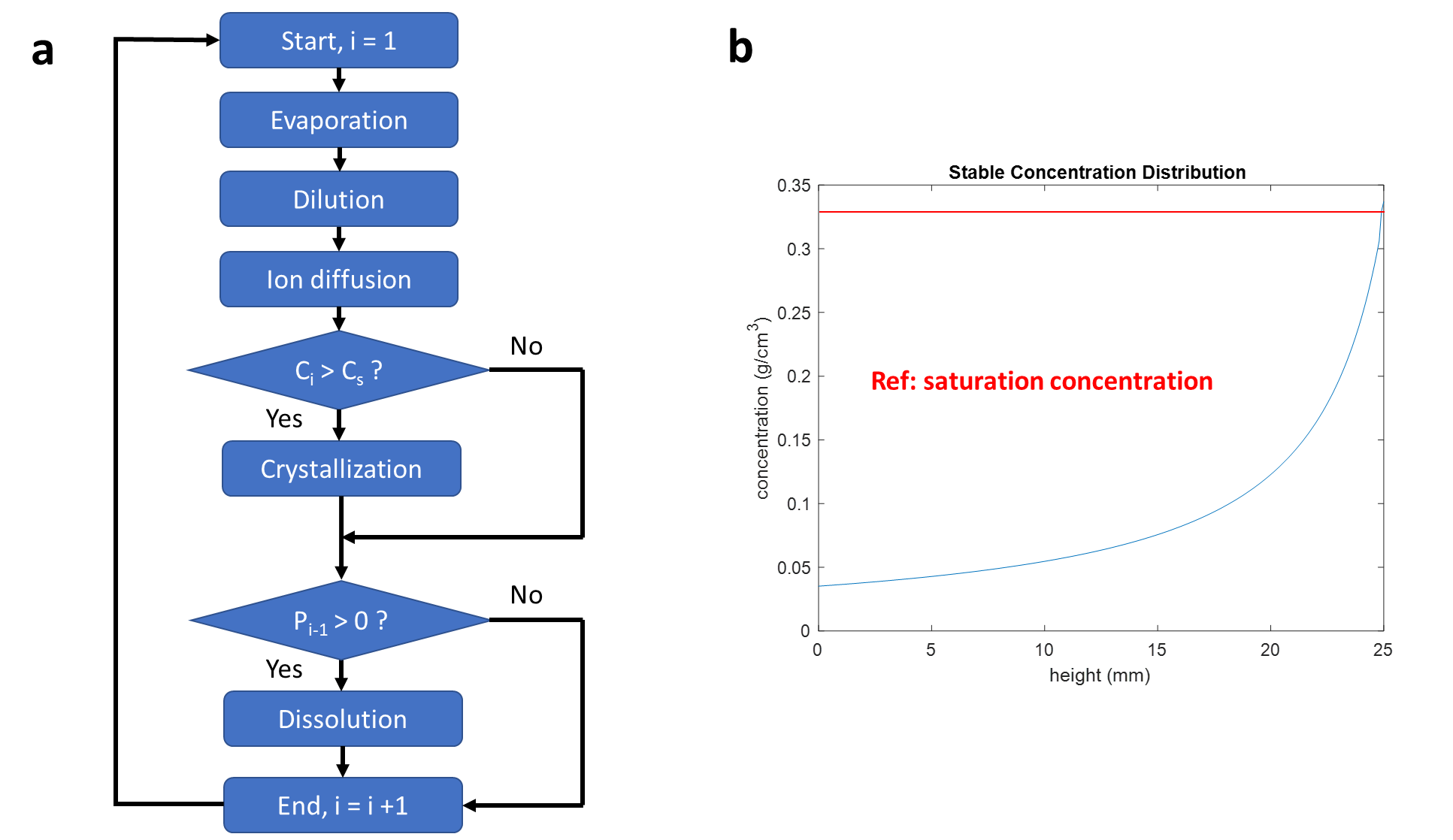
**

**Figure S24**: a) Flow chart of the calculation, b) Simulation result of stable concentration distribution along the capillary.

**Florescence imaging measuring the concentration gradient along the capillary**

Direct measurement of the concentration of salt water in the capillary is challenging due to the micro-scale dimension of the water in the capillary. To address this issue, Florescence imaging (Keyence BZ-X800 Epifluorence Microscope) is used to measure the concentration distribution along the capillary of SWBM. **Figure S25a** shows the experiment setup. Water with Rhodamine 6G (R6G) is constantly added at one end of SWBM (reservoir) and wicks its surface. R6G particles emit signal which is then collected by the objective below. The objective moves around to capture florescence images at different locations of the SWBM. The concentration of the R6G can be examined by looking at the distribution of the signal. Note that R6G is a water-soluble dye which behaves the same as the salt. Therefore, the concentration distribution of R6G inside the capillary is equivalent to the concentration distribution of the salt. Figure S24b is the stitched fluorescence image showing the concentration distribution of R6G over the SWBM. The concentration of R6G is low at the region close to the reservoir and high at the region away from the reservoir. The concentration along the capillary direction follows an exponential increase with the distance away from the reservoir (**Figure S25c**). Some much brighter spots on the passive region corresponds to the saturation concentration (crystallization) on the passive region. Therefore, the concentration of the solution exponentially increases along the capillary length and saturation concentration happens only at the passive region.

**
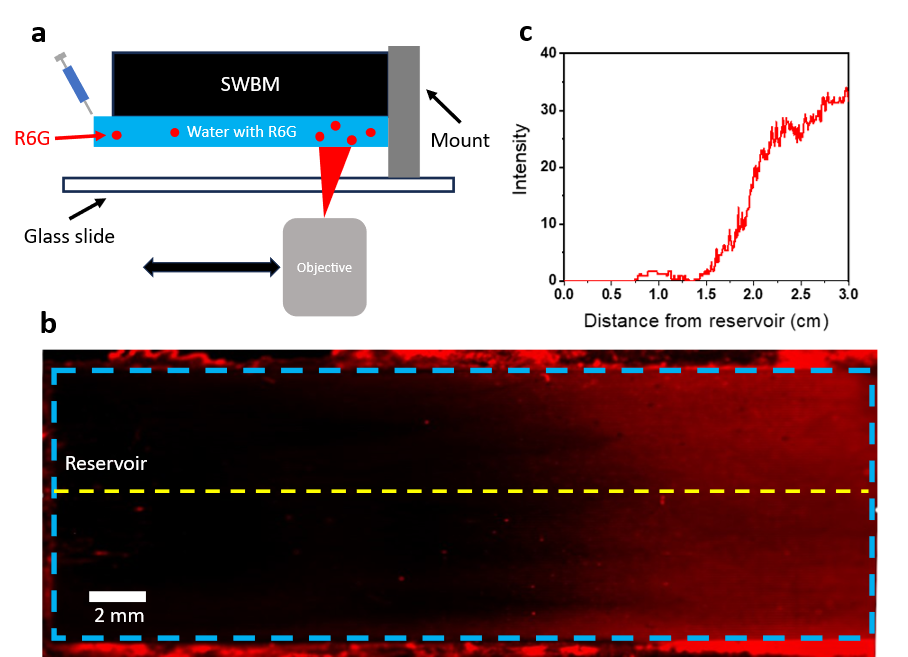
**

**Figure S25:** a) Florescence imaging setup using R6G. b) Fluorescence image of the SWBM. Blue dash line marks the active region of the SWBM, and yellow line marks the line for intensity analysis. c) R6G florescence signal intensity distribution along the capillary.

**Supplementary Note 15**

**Salt crystallization image and corresponding micro groove surface profile**

**
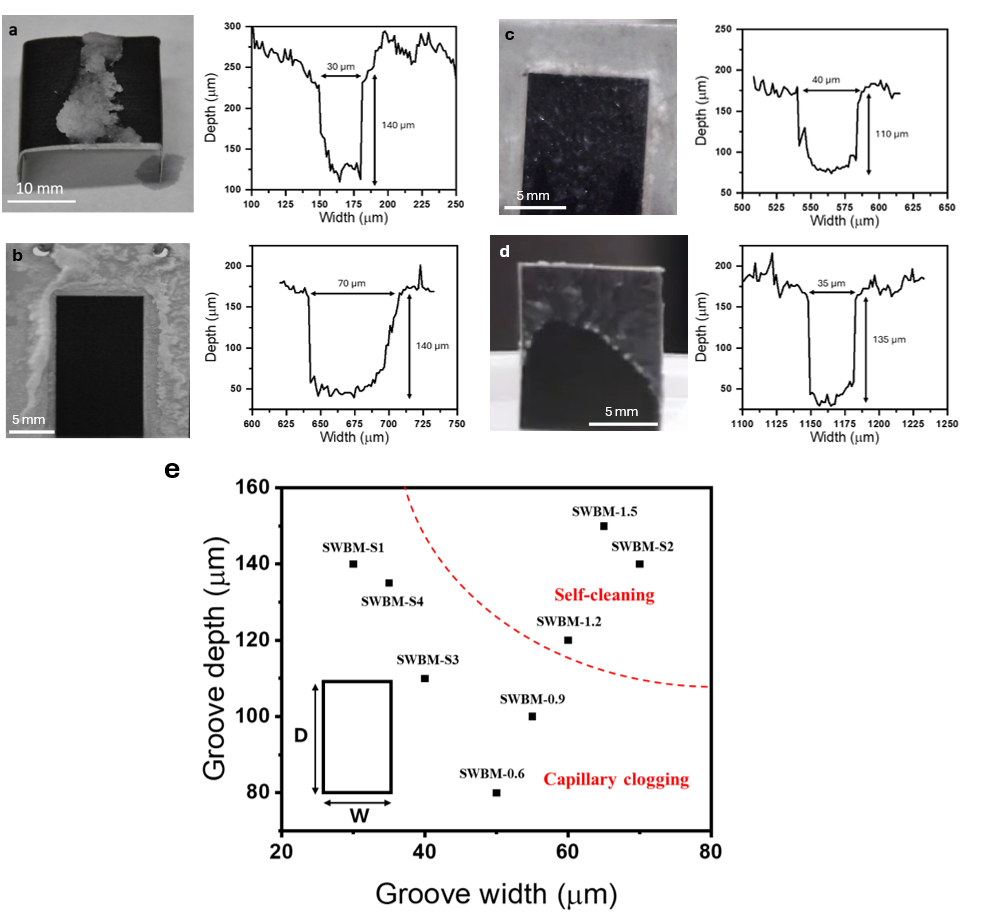
**

**Figure S26:** Salt crystallization image and corresponding micro groove profile for different SWBM, a) SWBM-S1: image reproduced from Singh et al., Nat Sustain (2020), licensed under CC BY 4.0 ^1^, the figure is original to the cited paper and does not contain third-party material requiring separate permission. b) SWBM-S2, c) SWBM-S3, d) SWBM-S4, e) Salt crystallization behavior for different SWBMs with different groove width and depth.

**Supplementary Note 16**

**Demonstration of Solar trackable ABF-STIC**

Solar tracking can be easily achieved by adjusting the inclination angle of the SWBM and the orientation of the SWBM. As shown in **Fig 6a**, ABF-STIC performance nearly doesn’t change at different inclination angles. Details of the amount of evaporation verse time and self-cleaning phenomenon at different inclination angle are shown in **Figure S27,28**. Therefore, ABF-STIC can track the sunlight to maximum solar flux and desalination performance. **Figure S28e** compares the amount of water desalinated between flat system and solar tracking system. The data was collected on Feb 21, 2021, on the roof of Wilmot Building of the Institute of Optics at University of Rochester, NY, USA (43.16° N, 77.61° W). Solar tracking provides a 77.4% enhancement for the desalination rate throughout the day.


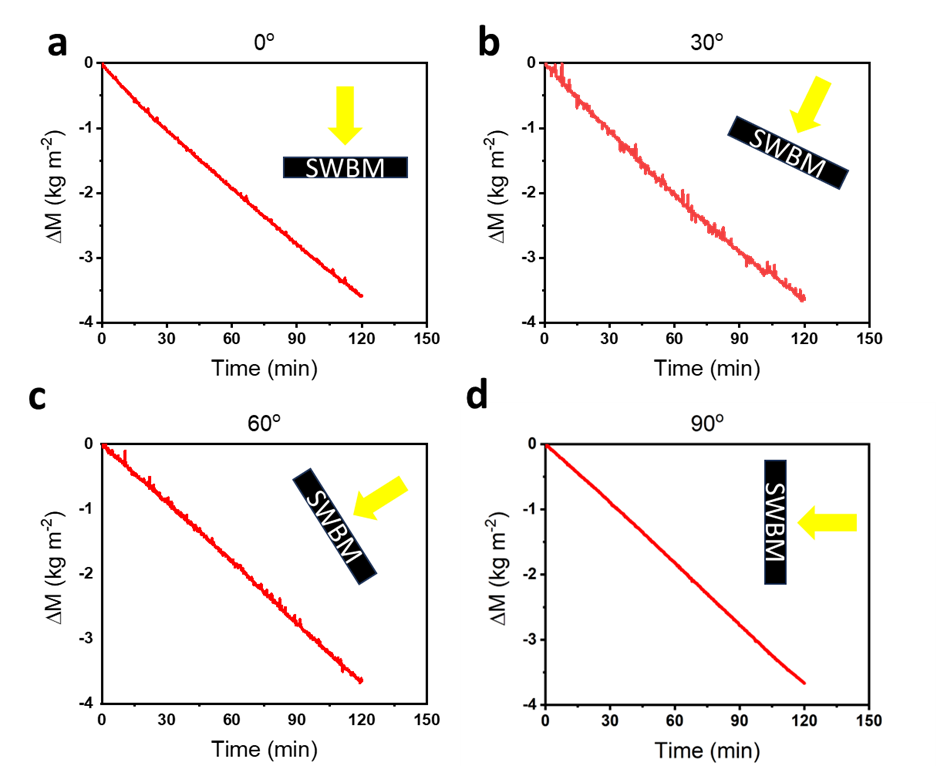


**Figure S27**: The amount of water evaporated verse time for SWBM placed at (a) 0, (b) 30, (c) 60 and (d) 90 degree inclination angle.


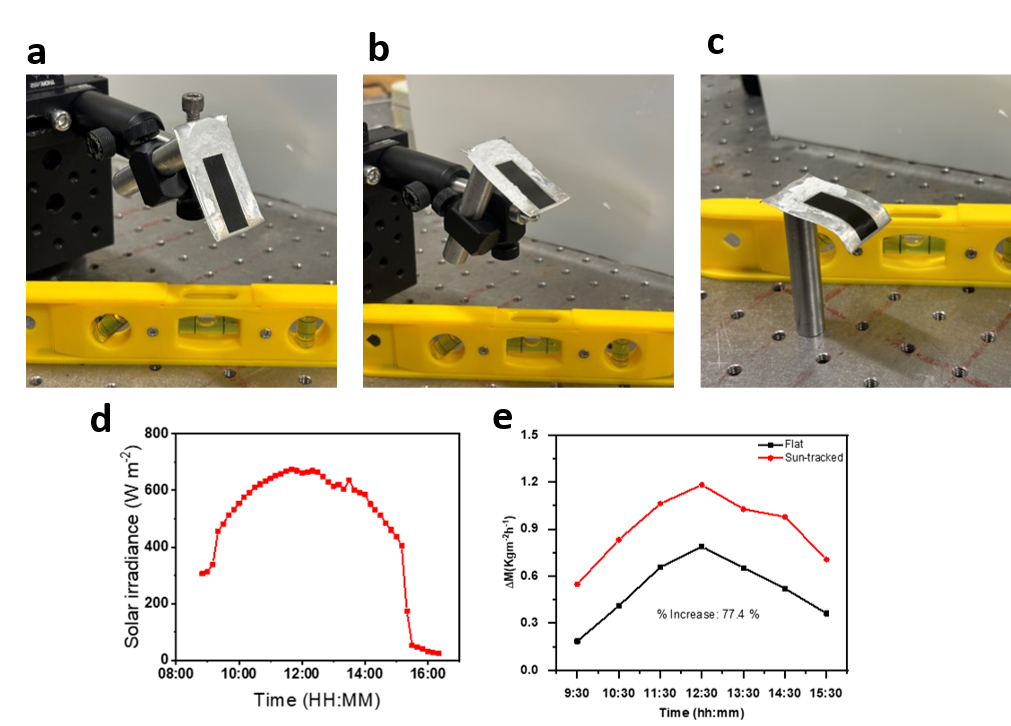


**Figure S28**: Self-cleaning SWBM when mounted at different inclination angle (a) 60, (b) 30, (c) 0 degree. (d) Solar irradiance during the outdoor experiment on Feb 21, 2021. (e) The rate of desalination comparison between flat system and solar tracking system.

**Implementation of solar tracking system and its economic benefits analysis**

**Fabrication of an automated dual-axis sun tracking system:**

A dual-axis sun-tracking solar-thermal desalination system, as shown in **Fig. S29**, can be fabricated using three servo motors, 2 pairs of optical sensors, and an Arduino microcontroller**.** A hemispherical dome can be used as a transparent window and condenser to condense water vapor into liquid water. A lightweight flat platform can be used to mount the ABF-STIC. Two light dynamics resistor (LDR) sensors (S1 and S2), mounted on both sides of the ABF-STIC and connected with motors M2 and M3, can track the azimuthal motion of the Sun. Similarly, a pair of LDR sensors (S3 and S4), mounted on the top and bottom of the platform and connected with a large servo motor (M1), can track the zenith motion of the Sun. The sensors and motors can be interfaced and programmed with an Arduino microcontroller.


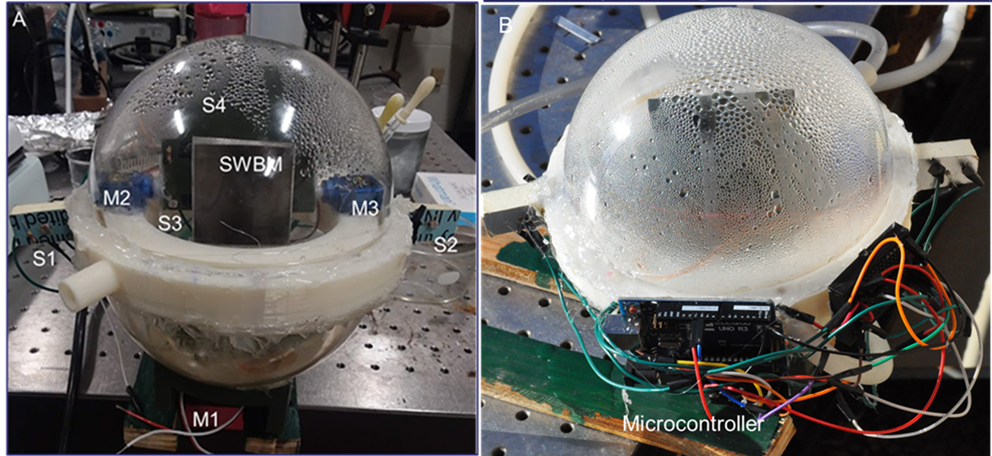


**Figure S29**: Dual-axis sun-tracking solar-thermal desalination system with (a) motors and sensors, (b) microcontroller.

**Feasibility:** The proposed system can be implemented on a seashore as well as in shallow regions of a sea.

**The initial cost of the solar tracking system**: The weight of a 1-m^2^ ABF-STIC made with a 0.2-mm thin Al sheet will be about 0.54 kg. A lightweight and corrosion-resistant insulating material, such as high-density (100 kg m^-3^) polyurethane foam, can be used as platform material. A 5-cm thick and 1.1 m×1.1 m large polyurethane platform (weight 6 kg) can be a suitable choice for the platform. The total weight of the DISC with the platform and sensors will be less than 10 kg. The servomotor M1 (price 15$) shown above can provide up to 22.5 kg-cm torque with 4.8 V DC input. The cost of an Arduino microcontroller is $10-20. LDR sensor cost is about $10 for 60-100 pieces. The total cost for 3 servomotors, and a microcontroller will be ~$60. Adding 15% overhead cost, the total initial investment could be ~$70.

Other parts, like condensers, are the same for both types of ABF-STIC systems.

**Operational cost:** A 20 V-100 W solar panel with a cost in the range of $75-100 can be used to power the tracker and the lifetime of the solar panel is ~ 25 yrs. Therefore, $3-4/year can be the operational cost of the tracker.

**Profit of additional water produced:** A 1-m^2^ flat-lying ABF-STIC generates ~ 10.33 liters of fresh water each day. A sun-tracked ABF-STIC can produce ~ 77 % (i.e. ~ 8 liters) of additional water over the horizontally lying panel (see **Supporting Information Fig. S28 (e))**. For an average of 200 days of annual operation, the Sun-tracked panel will produce ~ 1600 liters of extra fresh water per year. According to a 2022 market analysis, a 1.5-liter bottle of water from a local brand costs about $0.70 (0.46/liter). Based on this global average water price, the Sun-tracked panel will produce extra water priced ~ $736/year i.e. about $60/month.

**Payback period:** Total initial investment including the tracking system and solar panel will be ~$170 and the cost of the additional water produced ~$60/ month. Therefore, the pay-back period for the tracking system is ~ 3 months.

**Supplementary Note 17**

**Outdoor Experiment setup and product**

**
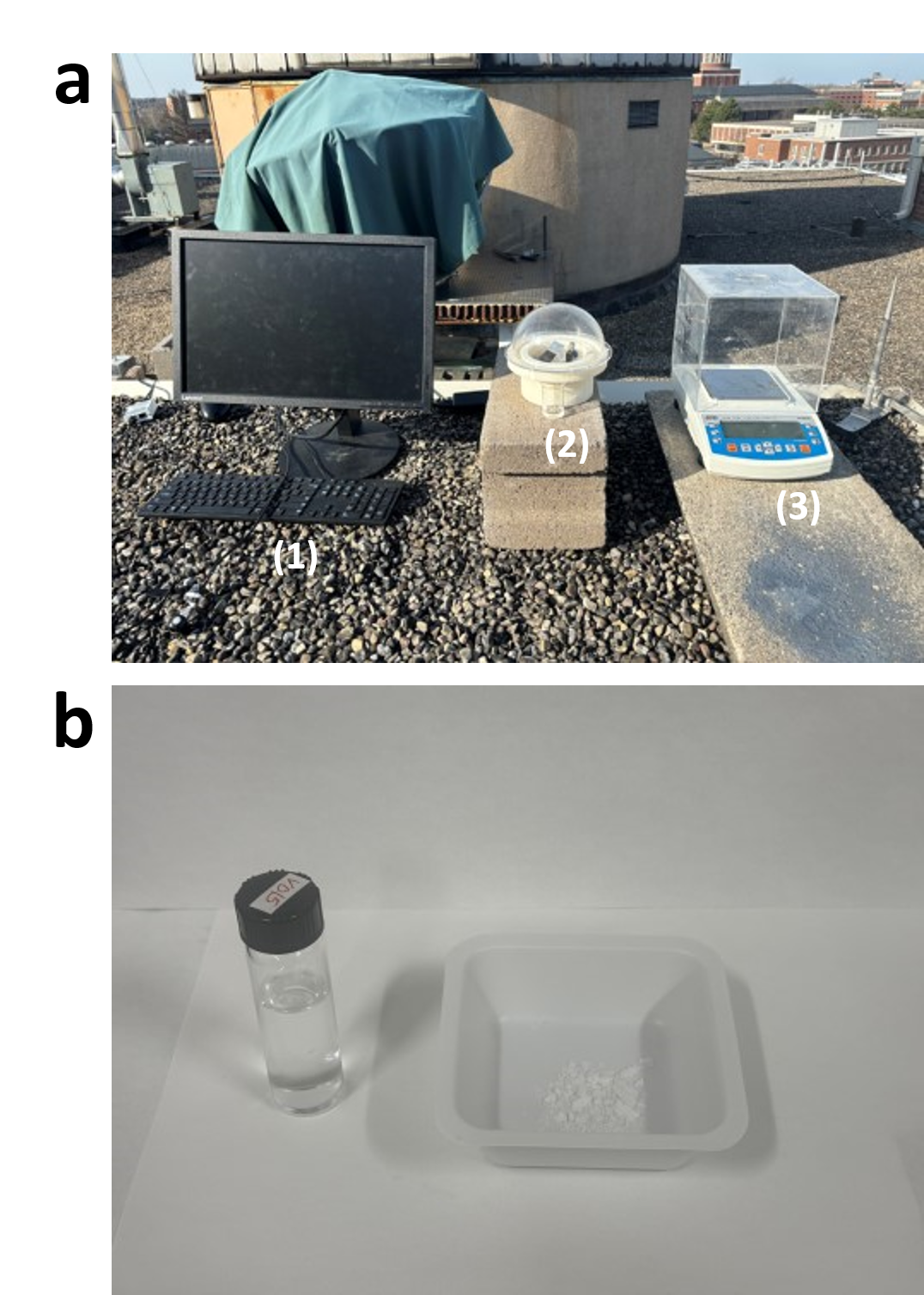
**

**Figure S30**: (a) Outdoor experiment Layout. (1) computer with sensor measuring solar irradiance, temperature and relative humidity, (2) ABF-STIC integrating with solar trackable condensation system, (3) balance measuring freshwater production rate. (b) Freshwater and seasalt harvested.

**Supplementary Note 18**

**Scalability analysis of the SWBM**

Scalability is important for real world applications. The scalability of the SWBM is analyzed in two directions: perpendicular to gravity (width) and against gravity (length). In the manuscript, the size of most of our studied SWBM samples is 3 cm x 1 cm (L x W).

The scaling in the width direction is analyzed by studying the performance of SWBM with sizes of 3 cm x 3 cm and 3 cm x 5 cm. **Figure S31a** displays the evaporation rate for SWBM of different sizes. The rate of evaporation increases linearly with the width of SWBM. Moreover, the surface salt rejection still preserves (**Figure S31c, d**). Therefore, SWBM is scalable in width. This is reasonable because the water transport rate is mainly determined by the characteristic of the microgrooves rather than the overall width of the SWBM.

To test the scalability in length direction, SWBM with a size of 9 cm x 1 cm and 15 cm x 1 cm is studied. **Figure S31b** shows the evaporation rate of the samples. The evaporation rate is also linearly proportional to the length of the sample with self-cleaning property (**Figure S31e**). However, this trend won’t hold infinity long due to gravity. **Figure S31f** displays the wicking dynamics of the sample. The water wicks very fast initially but then gradually slow down and will finally stop. For vertically mounted case, the maximum wicking length can be estimated with the Jurin’s Law:

$$\begin{aligned} L=\frac{\gamma\cos\left( \theta\right)}{\rho gr}=24.49 cm \#\left( 24 \right) \end{aligned}$$

where $\gamma$ is the surface tension of the liquid, $\theta$ is the contact angle of liquid on wall, $\rho$ is the density of the liquid and $r$ is the radius of the capillary.

Therefore, the scaling of the ABF-STIC in width direction is unrestricted and the scaling of the ABF-STIC in length reaches up to 15 cm which is unmatched by other existing system. This limit could be further alleviated by mounting the sample horizontally or using higher laser processing power in the higher part of the sample.

.
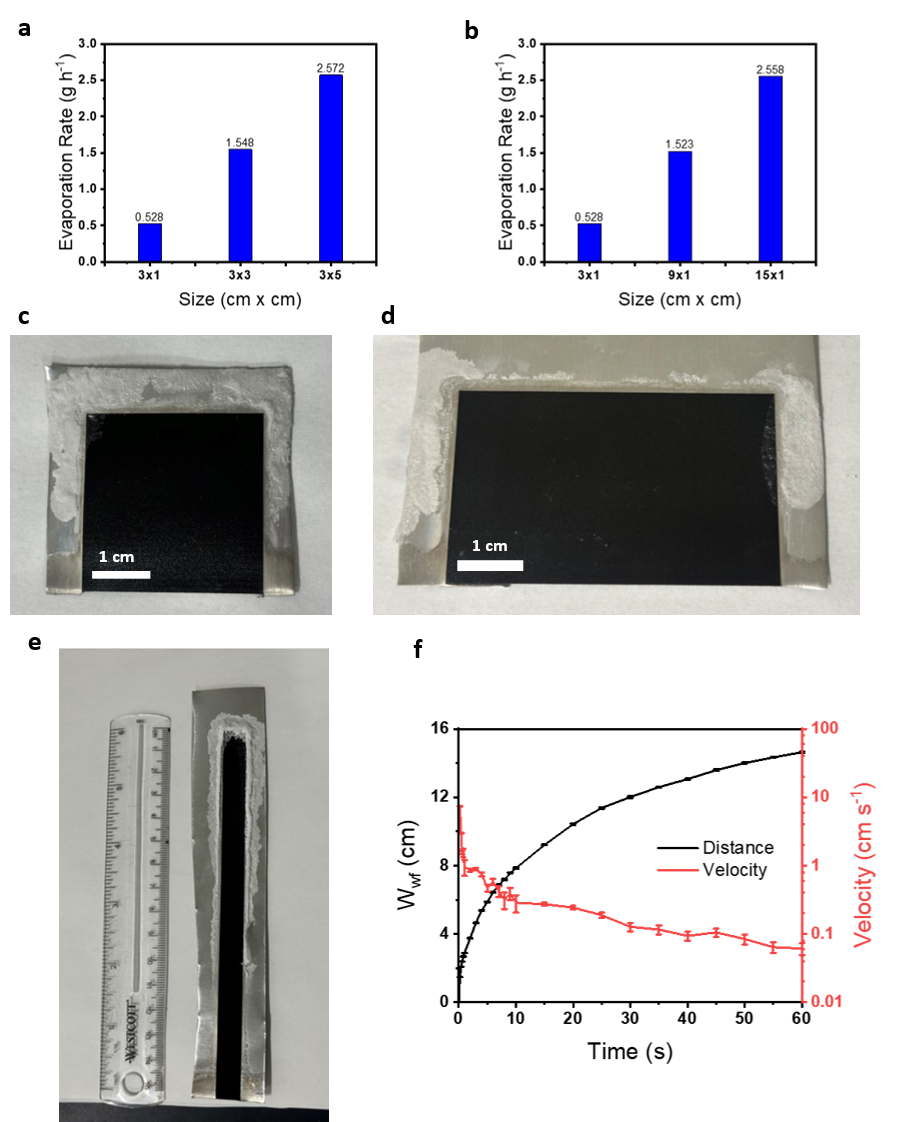


**Figure S31**: (a) the evaporation rate of SWBM when scaling the width. (b) the evaporation rate of SWBM when scaling the length. (c) self-cleaning SWBM with a size of 3 cm x 3cm. (d) self-cleaning SWBM with a size of 3 cm x 5 cm. (e) self-cleaning SWBM with a size of 15 cm x 1cm. (f) Water wet front and velocity verse time for 15 cm SWBM. Each error bar represents the standard deviation of mean calculated from at least 10 data points.

**Supplementary Note 19**

**Desalination test for different ocean water**

**
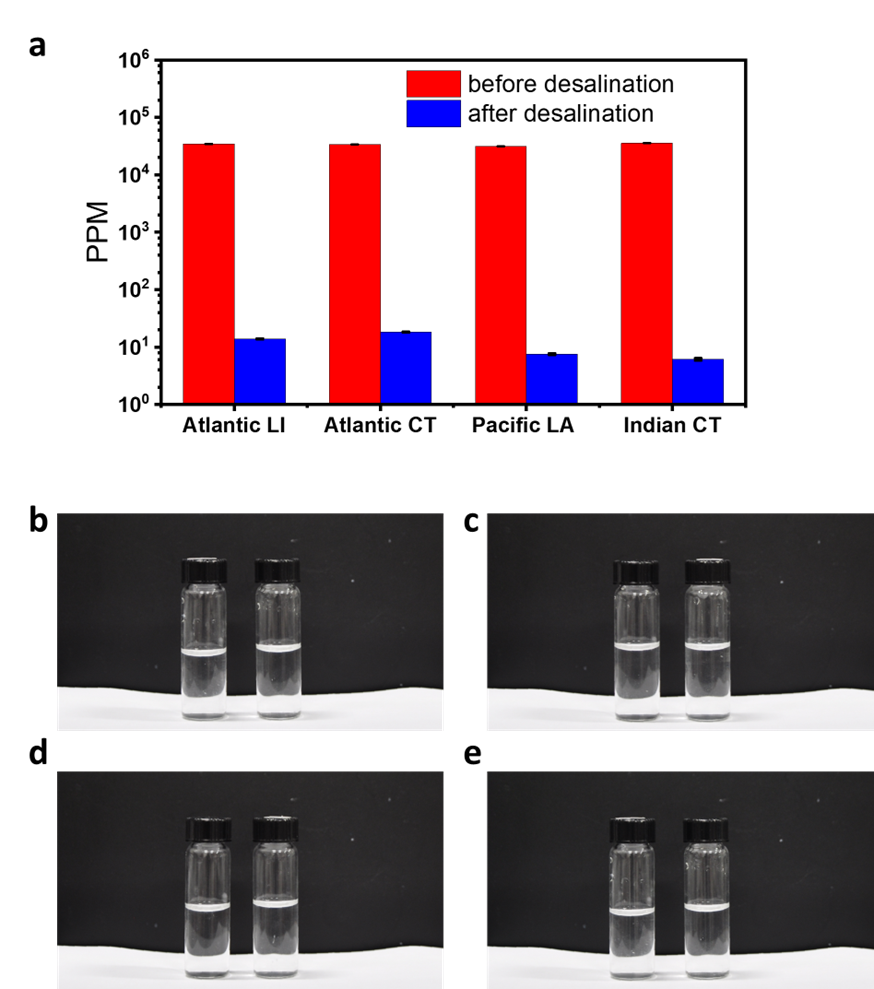
**

**Figure S32**: a) Salinity of different ocean water before and after desalination. (b-e) Photos of ocean water (left) and purified water (right) for different ocean, b) Atlantic Ocean from Fire Island, c) Atlantic Ocean from L’Agulhas, d) Indian Ocean from L’Agulhas, e) Pacific Ocean from Los Angeles.

**Captions for supplementary videos:**

**Supplementary Video 1**: a video presenting self-cleaning property of SWBM. The first 8 hours is in sun illumination and the rest 16 hours is in dark. The time is shown on the top left side and scale bar is shown on the bottom right side.

**Reference:**

1 Singh, S. C. *et al.* Solar-trackable super-wicking black metal panel for photothermal water sanitation. *Nature Sustainability* **3**, 938-946 (2020). <https://doi.org:10.1038/s41893-020-0566-x>

2 Jalil, S. A. *et al.* Spectral absorption control of femtosecond laser-treated metals and application in solar-thermal devices. *Light Sci Appl* **9**, 14 (2020). <https://doi.org:10.1038/s41377-020-0242-y>

3 Vorobyev, A. Y. & Guo, C. Femtosecond laser nanostructuring of metals. *Opt Express* **14**, 2164-2169 (2006). <https://doi.org:10.1364/oe.14.002164>

4 Singh, S. C. & Guo, C. Femtosecond laser‐produced optical absorbers for solar‐thermal energy harvesting. *EcoMat* **4** (2021). <https://doi.org:10.1002/eom2.12161>

5 Hertel, I. V., et al. "On the physics of material processing with femtosecond lasers." *Riken Review* (2001).

6 Shi, Y. *et al.* A 3D Photothermal Structure toward Improved Energy Efficiency in Solar Steam Generation. *Joule* **2**, 1171-1186 (2018). <https://doi.org:10.1016/j.joule.2018.03.013>

7 Zhang, C. *et al.* Designing a next generation solar crystallizer for real seawater brine treatment with zero liquid discharge. *Nat Commun* **12**, 998 (2021). <https://doi.org:10.1038/s41467-021-21124-4>

8 Nayar, K. G., Panchanathan, D., McKinley, G. H. & Lienhard, J. H. Surface Tension of Seawater. *Journal of Physical and Chemical Reference Data* **43** (2014). <https://doi.org:10.1063/1.4899037>

9 Sezer, B. *et al.* Detection and quantification of a toxic salt substitute (LiCl) by using laser induced breakdown spectroscopy (LIBS). *Meat Sci* **135**, 123-128 (2018). <https://doi.org:10.1016/j.meatsci.2017.09.010>

10 Davari, S. A., Taylor, P. A., Standley, R. W. & Mukherjee, D. Detection of interstitial oxygen contents in Czochralski grown silicon crystals using internal calibration in laser-induced breakdown spectroscopy (LIBS). *Talanta* **193**, 192-198 (2019). <https://doi.org:10.1016/j.talanta.2018.09.078>

11 Gondal, M. A. & Hussain, T. Determination of poisonous metals in wastewater collected from paint manufacturing plant using laser-induced breakdown spectroscopy. *Talanta* **71**, 73-80 (2007). <https://doi.org:10.1016/j.talanta.2006.03.022>

12 Sallé, B., Lacour, J. L., Mauchien, P., Fichet, P., Maurice, S. & Manhès, G. Comparative study of different methodologies for quantitative rock analysis by Laser-Induced Breakdown Spectroscopy in a simulated Martian atmosphere. *Spectrochimica Acta Part B: Atomic Spectroscopy* **61**, 301-313 (2006). <https://doi.org:10.1016/j.sab.2006.02.003>

13 Yaroshchyk, P., Morrison, R. J. S., Body, D. & Chadwick, B. L. Quantitative determination of wear metals in engine oils using LIBS: The use of paper substrates and a comparison between single- and double-pulse LIBS. *Spectrochimica Acta Part B: Atomic Spectroscopy* **60**, 1482-1485 (2005). <https://doi.org:10.1016/j.sab.2005.09.002>

14 Li, P. *et al.* Photoconversion of U(VI) by TiO2: An efficient strategy for seawater uranium extraction. *Chemical Engineering Journal* **365**, 231-241 (2019). <https://doi.org:10.1016/j.cej.2019.02.013>

15 Gibbs, W. E. & Clayton, W. The Production of Large, Clear, Cubical Crystals of Sodium Chloride. *Nature* **113**, 492-493 (1924). <https://doi.org:10.1038/113492c0>

16 Yong, Y., Lou, X., Li, S., Yang, C. & Yin, X. Direct simulation of the influence of the pore structure on the diffusion process in porous media. *Computers & Mathematics with Applications* **67**, 412-423 (2014). <https://doi.org:10.1016/j.camwa.2013.08.032>

17 Simon, B. Dissolution rates of NaCl and KCl in aqueous solution. *Journal of Crystal Growth* **52**, 789-794 (1981). <https://doi.org:10.1016/0022-0248(81)90377-8>
